# Retrospective review of growth in pediatric intestinal failure after weaning from parenteral nutrition

**DOI:** 10.1002/ncp.11209

**Published:** 2024-09-12

**Authors:** Anita M. Nucci, Hillary Bashaw, Alexander Kirpich, Jeffrey Rudolph

**Affiliations:** ^1^ Department of Nutrition Georgia State University Atlanta Georgia USA; ^2^ Division of Gastroenterology, Hepatology, and Nutrition Emory University School of Medicine Atlanta Georgia USA; ^3^ Department of Population Health Sciences, School of Public Health Georgia State University Atlanta Georgia USA; ^4^ Division of Pediatric Gastroenterology, Hepatology, and Nutrition UPMC Children's Hospital of Pittsburgh Pittsburgh Pennsylvania USA

**Keywords:** organ transplantation, parenteral nutrition, pediatrics, short bowel syndrome, teduglutide

## Abstract

**Background:**

Growth outcomes in children with intestinal failure (IF) after weaning from parenteral nutrition (PN) may be modified by primary diagnosis and interventions aimed at achieving enteral tolerance. We evaluated growth after weaning by diagnosis and intestinal transplant status and during treatment with the glucagon‐like peptide‐2 analog teduglutide.

**Methods:**

A two‐center retrospective review was conducted on children diagnosed with IF at age <12 months. The *z* scores for weight and length/height were examined up to 5 years after PN weaning and in children who received teduglutide for >6 months. Data were reported as median and interquartile range (IQR).

**Results:**

A total of 362 children (58% male and 72% White) were reviewed; 41% (*n* = 150) weaned from PN at age 1.5 years (IQR = 0.96–3). Weight and length/height data were available for 144 children; 46 received an intestinal transplant. Median weight and length/height *z* scores at weaning were −1.15 (IQR = −2.09 to −0.39) and −1.89 (IQR = −2.9 to −1.02), respectively. In those not transplanted, *z* scores remained stable (± 0.5 change). Children with small bowel atresia experienced accelerated linear growth (> +0.5 change) beginning in year 3. Most children transplanted experienced growth acceleration beginning in year 2. Fourteen children received teduglutide (median = 840 [IQR = 425–1530] days), and growth remained stable throughout treatment. Five were weaned from PN within 1 year.

**Conclusion:**

We observed stable growth with limited catch‐up after PN weaning, with minimal variation by diagnosis, and during teduglutide therapy. Children who received an intestinal transplant experienced acceleration in weight and linear growth after weaning.

## BACKGROUND

Intestinal failure (IF) in infants and children can occur as the result of a surgical resection or functional impairment of the small bowel and is a life‐threatening state characterized by malabsorption, malnutrition, and chronic growth failure.^1^ This condition is often the consequence of short bowel syndrome (SBS), in which adaptive responses of the intestine are at least initially insufficient to attain enteral autonomy. Morbidity and mortality rates in pediatric IF have been associated with multiple factors, including (1) age at the time of surgery, (2) residual bowel length, (3) presence or absence of the ileocecal valve (ICV), (4) function and adaptive capacity of the remnant bowel, (5) the ability to achieve enteral autonomy, (6) incidence of sepsis, and (7) the development of IF‐associated liver disease.^2^, ^3^ The inability to maintain growth is considered a primary indication for parenteral nutrition (PN) in the pediatric IF population. Interdisciplinary team management of IF can result in positive outcomes, including cessation of PN support, improved survival, and accelerated growth.^4^, ^5^

Despite the advancement in intestinal rehabilitation (IR) care, many children still fail to maintain adequate somatic growth.^6^, ^7^, ^8^, ^9^ Infants with IF have been observed to have an appropriate birth weight and length for gestational age followed by a decline during the first 6 months of life regardless of PN status.^8^, ^10^, ^11^ A subsequent rebound in growth parameters has been reported by some centers for IR during year 2.^10^, ^12^ Older children and adolescents with IF often have growth parameters significantly lower than reference values from the general population^13^, ^14^, ^15^ and children diagnosed with other gastrointestinal motility disorders.^16^ Earlier studies have reported adequate growth in children who were receiving full PN.^17^, ^18^ Linear growth velocity, however, has been found to decline in the early years after PN weaning.^9^, ^17^, ^19^ Maintaining adequate growth following pediatric intestinal transplant can be challenging owing to episodes of increased output that result in the reduction of enteral support or occurrences of rejection that may require the administration of corticosteroids.^20^, ^21^ The results of previous research on growth after pediatric intestinal transplant vary. Some centers have reported growth maintenance with no/limited catch‐up growth^22^, ^23^, ^24^ whereas others observed accelerated linear growth.^25^ To summarize, although care has improved over the years in both the nutrition and medical management of the IF population, concern surrounding adequate growth outcomes has remained.

The most effective management strategy for stimulating intestinal adaptation, achieving IR, and reducing the risk of PN‐related complications has been the provision of enteral nutrition support.^3^, ^26^ However, children with SBS require months to years to adapt, and still others are never able to achieve enteral autonomy.^2^, ^4^ We do not yet fully understand the confluence of factors, such as diagnosis, intestinal anatomy, mode of nutrition intake, or initial growth status, that contribute to the problem within this population. Glucagon‐like peptide‐2 (GLP‐2) is an intestinotrophic hormone that, in response to food ingestion, increases blood flow to the gastrointestinal tract and activates pathways in the gut to enhance nutrient absorption.^27^ Treatment with a GLP‐2 analog (teduglutide) has been found to reduce PN volume and infusion dependency in children with SBS in clinical trials.^28^, ^29^, ^30^ No significant change in growth trajectory over the intervention periods has been reported.

Maintenance of adequate growth during IR and after enteral autonomy is achieved remains a challenge for many children with IF and their healthcare providers. Although growth patterns in this population have been reported previously and are relatively consistent, it is not known whether factors specific to either the primary cause of the IF or the strategies involved in treating the IF play a role. Using existing registries approved by institutional review boards (IRBs) at two centers for IR in the United States, our objectives were to conduct a retrospective multicenter observational study to (1) describe the pattern of growth in children with IF after weaning from PN and after subdivision by primary diagnosis and intestinal transplant status and (2) describe growth in children who received the GLP‐2 analog teduglutide. Ultimately, a better understanding of long‐term growth trajectory in the heterogenous pediatric IF population through acknowledgment of disease‐ and intervention‐specific differences will aid in the development of a treatment approach and improve growth outcomes for these children.

## METHODS

### Participants

This retrospective observational study was conducted at two centers for pediatric IR in the United States. The Intestinal Care & Rehabilitation Center (ICARE) at the UPMC Children's Hospital of Pittsburgh, Pittsburgh, PA has served as a regional, national, and international center for the evaluation and management of children with SBS/IF since the 1980s. The Intestinal Rehabilitation of Children (IROC) at Children's Healthcare of Atlanta, Atlanta, GA is a local and regional center for the treatment of children with SBS/IF that was established in 2010. The centers are staffed by an interdisciplinary team of pediatric specialists including a gastroenterologist, pediatric surgeon, transplant surgeon, clinical dietitians, and nurse practitioner. In general, the children were followed up every 4 to 12 weeks. Energy requirements for children receiving PN were determined using the Schofield equation.^31^ Protein requirements were individualized based on age and organ function. After PN weaning, energy and nutrient requirements were modified based on growth and nutrition laboratory monitoring. Enteral support was continued after PN weaning as necessary to meet nutrition requirements.

The eligibility criteria included diagnosis with IF (PN use >60 days within a 74 consecutive day interval) before age 12 months.^26^ Participants include patients who were referred to ICARE (*n* = 321) or IROC (*n* = 41) for IR between September 1989 and January 2023. Only children who were not receiving PN at the time of data collection and had weight and length/height data available at the time of PN weaning were included in the analysis. If PN had been stopped and then restarted (*n* = 10) the child was excluded. Given the advances in medical and surgical treatments for pediatric IF since the 1980s, we subdivided the cohort into two time periods (1989–2012 or 2013–2023) during analysis. The year of diagnosis periods were determined based on the publication of the initial report of the Pediatric IF Consortium (PIFCon) in 2012.^2^ Gattini et al noted that the medical, surgical, and nutrition management of children with IF improved after the PIFCon publication.^32^ The study was approved by the IRBs at the University of Pittsburgh, Children's Healthcare of Atlanta, and Georgia State University.

### Variables

Demographic characteristics, initial diagnosis, intestinal anatomy (small bowel length, percentage small bowel remaining after initial surgery, bowel‐lengthening procedures, presence/absence of an ICV, and area of large intestine), anthropometric data, mode of nutrition therapy, transplant status, and use of teduglutide were extracted from existing IRB‐approved ICARE and IROC registries. The percentage of small bowel remaining after initial surgery was estimated using normal values for intestinal length by gestational age.^33^ Anthropometric and nutrition therapy data were collected quarterly during the first year of follow‐up and biannually thereafter. Weight (kilograms) was measured with a digital medical scale. Height (centimeters) was determined using a stadiometer. Infants and toddlers (newborn to 24 months) had their weight and length (centimeters) measured using a digital infant scale and recumbent length board. The *z* score values for weight and length/height and body mass index (BMI) are reported for the total population and after subdivision by primary diagnosis (midgut volvulus, necrotizing enterocolitis (NEC), small bowel atresia, gastroschisis, Hirschsprung disease, microvillus atrophy, megacystis microcolon intestinal hypoperistalsis syndrome [MMIHS] or microvillus inclusion disease, and tufting enteropathy), intestinal transplant status, and year of diagnosis period in those who weaned from PN and were not restarted on PN during the review period. Anthropometric values are also reported for those who received a GLP‐2 analog (0.05 mg/kg/day teduglutide) for >6 months (2017–2023). Children who received teduglutide were targeted to be monitored every 1–2 months after the initiation of therapy and at a minimum of every 6 months thereafter.

The *z* score statistics for weight, length/height, and BMI were determined using age‐ or gestational age–adjusted and sex‐appropriate growth charts from Fenton et al (2013)^34^ for preterm infants, the World Health Organization^35^ for infants and children age 0–2 years, and the Centers for Disease Control and Prevention (2000)^36^ for children >2 years. Achievement of enteral autonomy was defined as the maintenance of normal growth (± 0.5 *z* score) and hydration status using enteral support (tube feeding or oral diet) without the use of parenteral support (fluids containing any combination of macronutrients, electrolytes, vitamins, or trace elements) *for a period of >3 consecutive months*.

### Statistical analysis

Frequency analysis was conducted on all demographic and clinical variables. Testing for normality was conducted on all continuous variables. Mean ± SD was used to describe variables that were normally distributed. Median and interquartile range (IQR) was used to describe variables with a skewed distribution. For children who weaned from PN and did not resume PN during the study period, anthropometric values (*z* scores) were plotted by day of life from the time of the first visit to the last interaction. Summary statistics for weight, length/height, and BMI were calculated for the entire sample of children who weaned from PN and did not resume PN during the study period as well as by primary diagnosis, intestinal transplant status (no intestinal transplant or intestinal transplant), and year of diagnosis category from the time of PN discontinuation (baseline) up to 5 years after weaning (time points: 30, 60, 90, 120, 180, 360, 540, 720, 900, 1080, 1260, 1440, 1620, and 1800 days). Corresponding *z* score values were extrapolated from anthropometric measurements taken before and after each time point. The change in median anthropometric *z* score was calculated in relation to the time of PN discontinuation (baseline) and extrapolated measurement for each follow‐up time point for children weaned from PN. For all participants who received teduglutide, measured anthropometric values (*z* scores) were also plotted by day of life starting up to 2 years before medication started and up to 3 years after the initiation of therapy. The change in *z* scores was defined as decelerating (>−0.5), stable/maintained (±0.5), or accelerating (>+0). The Mann‐Whitney *U* test was used to evaluate differences in the age of PN weaning by diagnosis and intestinal transplant status, weight and length/height by diagnosis, intestinal transplant status, and time period, as well as years receiving PN and total bilirubin by time period. All statistical analyses were performed using the R programming language (v4.2.1) and RStudio environment (v2023.09.01).

## RESULTS

### Demographic and clinical characteristics

Demographic and clinical characteristics of the population are shown in Table 1. The combined registries included 362 children with IF (58% male and 72% White) with a median age at diagnosis of 6 days (IQR = 1–22). Common primary diagnoses included NEC (27%), gastroschisis (23%), and small bowel atresia (16%). The median gestational age was 34 weeks (IQR = 31–37), median length of small bowel remaining after initial surgery or diagnosis was 26 cm (IQR = 15–45), and median percentage of small bowel remaining after initial surgery or diagnosis was 23% (IQR = 10–50). In those for whom the status of the ICV and colon were known, ~37% (*n* = 130) had an ICV and ~49% (*n* = 161) had a full colon. The median follow‐up time was 6.4 years (IQR = 4.2–7.3). A total of 212 of the 362 children (~59%) did not wean from PN or their PN wean status was unknown. Of the 212, 59 were actively receiving care by a center for IR and remained on PN, 97 died, and 56 were lost to follow‐up. Approximately 41% of children (*n* = 150) were successfully weaned from PN. The median time receiving PN was 1.4 years (IQR = 0.9–2.8). Of these, 46 had received an intestinal transplant (12 isolated intestine, 21 liver/intestine, and 13 multiorgan). The majority of children who weaned from PN achieved enteral autonomy (*n* = 133, 40 after intestinal transplantation). The remaining 16 either did not maintain normal growth, remained on intravenous fluid, or were lost to follow‐up. The median age at the time of PN weaning was 1.5 years (IQR = 0.96–3). Children who received an intestinal transplant weaned from PN a median of 1 month (IQR = 0–2.5) after transplant. The demographic characteristics of the population of children who received teduglutide (*n* = 14) are shown in Table 2. The demographic characteristics were similar to the overall population. However, the median percentage of small bowel remaining at the time of diagnosis and the total bilirubin at the initial visit were lower at 10% (IQR = 9–16) and 3.5 mg/dl (IQR = 2.2–7), respectively.

**Table 1:** Demographic and clinical characteristics of the ICARE and IROC populations.

	Total	ICARE	IROC
	(*N* = 362)	(*N* = 321)	(*N* = 41)
Sex, *n* (%)			
Male	209 (57.7)	189 (58.8)	20 (48.8)
Female	153 (42.3)	132 (41.2)	21 (51.2)
Gestational age, median (IQR), weeks	34 (31–37)	35 (31–37)	34 (27.5–36)
Race, *n* (%)			
White	260 (71.8)	242 (75.4)	18 (43.9)
African American	70 (19.3)	47 (14.6)	23 (56.1)
Asian	4 (1.1)	4 (1.2)	0 (0)
Native Hawaiian/other Pacific Islander	1 (0.3)	1 (0.3)	0 (0)
Other	10 (2.8)	10 (3.1)	0 (0)
Missing	17 (4.7)	17 (5.3)	0 (0)
Primary diagnosis, *n* (%)			
Surgical			
Necrotizing enterocolitis	99 (27.3)	85 (26.5)	14 (34.1)
Gastroschisis	82 (22.7)	71 (22.1)	11 (26.8)
Small bowel atresia	63 (17.4)	56 (17.5)	7 (17.1)
Midgut volvulus	35 (9.7)	35 (10.9)	0 (0)
Omphalocele	3 (0.8)	3 (0.9)	0 (0)
Colonic atresia	1 (0.3)	0 (0)	1 (2.4)
Functional			
Hirschsprung disease	24 (6.6)	22 (6.9)	2 (4.9)
Microvillus atrophy	9 (2.5)	9 (2.8)	0 (0)
MMIHS or microvillus inclusion disease	13 (3.6)	12 (3.7)	1 (2.4)
Pseudo‐obstruction	6 (1.7)	6 (1.9)	0 (0)
Tufting enteropathy	4 (1.1)	3 (0.9)	1 (2.4)
Other	20 (5.5)	16 (5.0)	4 (9.8)
Missing	3 (0.8)	3 (0.9)	0 (0)
Small bowel remaining at diagnosis, median (IQR), %	23 (10.5–50)	22 (10–50)	25 (12.1–41.5)
Presence of ileocecal valve, *n* (%)^a^	130 (37.4)	119 (38.7)	11 (26.8)
All colon remaining, *n* (%)^a^	161 (48.9)	149 (51.3)	12 (30.8)
Total bilirubin at initial visit, median (IQR), mg/dl	6.2 (2.5–11)	6.8 (3–11.6)	3.6 (0.9–6.9)
Weaned from parenteral nutrition, *n* (%)	150 (41.4)	131 (40.8)	19 (46.3)
Achieved enteral autonomy, *n* (%)	133 (36.7)	114 (35.5)	19 (46.3)
Small bowel transplant, *n* (%)	73 (20.2)	72 (22.4)	1 (2.4)
Died, *n* (%)	106 (29.3)	106 (33)	0 (0)

Abbreviations: ICARE, Intestinal Care & Rehabilitation Center; IROC, Intestinal Rehabilitation of Children; IQR, interquartile range; MMIHS, megacystis microcolon intestinal hypoperistalsis syndrome.

^a^ Based on the number of children with a known status for ileocecal valve (ICARE = 307 and IROC = 41) and colon (ICARE = 290 and IROC = 39) at the initial visit.

**Table 2:** Demographic and clinical characteristics of children who received teduglutide.

	Total^a^
	(*N* = 14)
Sex, *n* (%)	
Male	9 (64.3)
Female	5 (35.7)
Gestational age, median (IQR), weeks	33 (30.5, 35.5)
Race, *n* (%)	
White	11 (78.6)
African American	3 (21.4)
Primary diagnosis, *n* (%)	
Gastroschisis	6 (42.9)
Necrotizing enterocolitis	4 (28.6)
Midgut volvulus	3 (21.4)
Other	1 (7.1)
Small bowel remaining at diagnosis, median (IQR), %	10 (9, 16)
Presence of ileocecal valve, *n* (%)^b^	5 (35.7)
All colon remaining, *n* (%)^b^	9 (64.2)
Age at initiation of teduglutide, median (IQR), years	9.5 (5.8, 11)
Total bilirubin at initial visit, median (IQR), mg/dl	3.5 (2.2, 7)
Weaned from parenteral nutrition, *n* (%)	3 (21.4)
Achieved enteral autonomy	2 (14.3)

^a^ Received teduglutide ≥6 months (all followed by the Intestinal Care & Rehabilitation Center at UPMC Children's Hospital of Pittsburgh, Pittsburgh, PA).

^b^ Based on the number of children with known status for ileocecal valve and colon.

### Growth after PN weaning

Weight and length/height values were available after PN weaning for 144 children. The primary diagnosis, intestinal transplant status, and diagnosis year for these children is shown in Table 3. The median age of PN weaning differed significantly by primary diagnosis. The median age of PN weaning in the primary diagnosis groups ranged from 412 to 884 days, except for children with MMIHS or microvillus inclusion disease who weaned at a median of 1240 days and those with pseudo‐obstruction who weaned much later at a median of 2912 days (*P* < 0.05). The median *z* score for weight and length/height at the time of PN weaning did not differ by primary diagnosis. The median age of PN weaning did not differ significantly in those who did not receive a transplant (496 days [IQR = 302–1221]) vs those transplanted (595 days [IQR = 446–861]). Although the *z* score for weight did not differ in those not transplanted (−0.86 [IQR = −2.07 to 0.07] vs those transplanted (−1.26 [IQR = −2.28 to −0.57]), the median *z* score for length/height was higher in children who did not receive a transplant (−1.44 [IQR = −2.66 to −0.33] vs −2.68 [IQR = −3.85 to −1.25], respectively).

**Table 3:** The number of children weaned from parenteral nutrition by primary diagnosis, intestinal transplant status, and time period of diagnosis.

Diagnosis	No intestinal transplant, *n*			Intestinal transplant, *n*
	Total	Diagnosis year	Diagnosis year	Total diagnosis year
		1989–2012	2013–2023	1989–2012
All diagnoses	98	63	35	46
Necrotizing enterocolitis	42	27	15	5
Gastroschisis	13	9	4	11
Small bowel atresia	20	9	11	6
Midgut volvulus	10	9	1	10
Hirschsprung disease	2	1	1	2
Microvillus atrophy	0	0	0	4
MMIHS or microvillus inclusion disease	0	0	0	2
Pseudo‐obstruction	0	0	0	1
Tufting enteropathy	0	0	0	1
Other	11	7	4	1
Missing	0	0	0	3

Abbreviation: MMIHS, megacystis microcolon intestinal hypoperistalsis syndrome.

Median weight, length/height, and BMI *z* score values at baseline and up to 1800 days (~5 years) after PN was successfully weaned in the total population and after subdivision by intestinal transplant status are shown in Table S1A–C. Median weight and length/height *z* scores at weaning for the total population were −1.15 (IQR = −2.09 to −0.39) and −1.89 (IQR = −2.9 to −1.02), respectively. For those who were not transplanted, weight gain and linear growth velocity were maintained throughout the follow‐up period with a deceleration of BMI beginning at day 1440 (~4 years). In those who received an intestinal transplant, catch‐up growth for weight and length/height (>+0.5 change in *z* score) was observed beginning at day 900 (~2.5 years) and day 720 (~2 years) after PN wean, respectively, with a stable BMI trajectory.

Median *z* scores for weight, length/height, and BMI at each time point for each diagnosis and after subdivision by transplant status are shown in Tables S2–S10. In children who did not receive an intestinal transplant, those with midgut volvulus and gastroschisis experienced deceleration (>−0.5 change in *z* score) in weight beginning in year 3 after PN wean and deceleration in length/height and BMI beginning in year 4. Children with NEC were observed to have stable gain (±0.5 change in *z* score) in weight and length/height over the approximately 5‐year follow‐up period, whereas children with small bowel atresia experienced stable gain in weight and accelerated linear growth velocity beginning at day 1080 (3 years) after PN wean. Two children with Hirschsprung disease were observed to have early acceleration (beginning in year 1) in both weight and length/height.

In children who received an intestinal transplant, the majority experienced acceleration in weight gain and linear growth after weaning from PN. Acceleration in weight gain occurred early in children with NEC (60 days) and microvillus atrophy (180 days) and later in children with Hirschsprung disease (1080 days or ~3 years). Catch‐up linear growth began as early as 90 days in children with NEC and MMIHS or microvillus inclusion disease, 540 days (~1.5 years) in children initially diagnosed with midgut volvulus, small bowel atresia, gastroschisis, and microvillus atrophy, and as late at 1440 days (~4 years) in one child with pseudo‐obstruction. Conversely, two children with Hirschsprung disease experienced a deceleration in linear growth beginning at 540 days (~1.5 years) after PN wean.

### Growth during GLP‐2 analog therapy

Fourteen children received a GLP‐2 analog for at least 6 months with a median treatment time of 840 days (IQR = 425–1530). The median age of therapy initiation was 9.5 years (IQR = 5.8–11). Five children (~36%) were weaned from parenteral support during the follow‐up period. Two (14%) were weaned within 6 months of starting treatment and of these, one achieved enteral autonomy. The other three children were weaned from PN between 6 and 12 months of treatment and two achieved enteral autonomy. A sixth child weaned from PN 20 months after therapy started but resumed PN for 5 months because of poor growth. This child has recently been weaned from PN. The pattern of growth of the teduglutide cohort is shown in Figure S1A–C. Median weight *z* score and length/height *z* score at the time of initiation of teduglutide were −2.38 (IQR = −2.99 to −1.41) and −2.99 (IQR = −3.57 to −1.8), respectively. Changes in weight and linear growth velocity *z* scores between baseline and up to 3 years after initiation are shown in Table 4. Most children who received teduglutide experienced maintenance or acceleration in their weight trajectory during the 3‐year follow‐up period with ~40% of children experiencing accelerated weight gain in the first 6 months of treatment. Linear growth velocity was stable for the majority of children through 3 years after the initiation of teduglutide. However, 31% of children experienced catch‐up linear growth between 6 months and 1 year after treatment start. One child was an intestinal transplant recipient who lost the graft because of rejection and subsequently resumed PN. Of the five children who weaned from PN, three (primary diagnoses of midgut volvulus, NEC, and gastroschisis) had stable weight gain and accelerated linear growth after weaning. One child with NEC experienced a deceleration of both weight and linear growth, and one child with gastroschisis weaned too recently to evaluate outcomes since weaning.

**Table 4:** Change in weight and length/height *z* score during GLP‐2 analog treatment in children with intestinal failure.

Time after GLP‐2 analog start	*N* a	Deceleration (*z* score change >−0.5), *n* (%)	Maintenance (*z* score change ±0.5), *n* (%)	Acceleration (*z* score change >+0.5), *n* (%)
Weight				
Start–6 months	13	0 (0)	8 (61)	5 (39)
6 months–1 year	13	1 (8)	10 (77)	2 (15)
1 year–1.5 years	10	3 (30)	7 (70)	0 (0)
1.5 years–2 years	7	0 (0)	7 (100)	0 (0)
2 years–2.5 years	6	2 (33)	3 (50)	1 (17)
2.5 years–3 years	5	2 (40)	1 (20)	2 (40)
Length/height				
Start–6 months	13	1 (8)	11 (84)	1 (8)
6 months–1 year	13	1 (8)	8 (61)	4 (31)
1 year–1.5 years	10	1 (10)	9 (90)	0 (0)
1.5 years–2 years	7	0 (0)	7 (100)	0 (0)
2 years–2.5 years	6	0 (0)	5 (83)	1 (17)
2.5 years–3 years	5	2 (40)	3 (60)	0 (0)

Abbreviation: GLP‐2, glucagon‐like peptide‐2.

^a^ Initial weight and length/height available for 13 of 14 children.

### Year of diagnosis

Given the evolution of care over the period examined, we divided the data into two eras (1989–2012 vs 2013–2023). The majority of children in the registries were diagnosed in the earlier time period (*n* = 279) in which the rate of PN weaning was 39.8%. In the later time period (*n* = 83), the rate of PN weaning was 45.8%. The median number of years receiving PN differed significantly between the 1989–2012 and 2013–2023 time periods (1.6 years [IQR = 1.1–3.5] vs 1.1 years [IQR = 0.6–1.9]; *P* < 0.001) as did the initial visit total bilirubin level (7.5 mg/dl [IQR = 3.6–12.7] vs 4.2 mg/dl [IQR = 0.9–6.8]; *P* < 0.001). All children who received an intestinal transplant were diagnosed before 2012. Therefore, no comparison of anthropometrics at the time of PN weaning by time period was made for those who were transplanted. For those who did not receive an intestinal transplant, no statistically significant difference was found in median weight *z* score at the time of PN weaning in those who were diagnosed between 1989–2012 (−0.84 [IQR = −2.33 to −0.3]) and 2013–2023 (−1.04 [IQR = −1.91 to −0.18]) or in median height *z* score (−1.62 [IQR = −2.6 to −0.51] vs −1.26 [IQR = −2.84 to −0.19]).

## DISCUSSION

This study examined growth patterns after discontinuation of PN in a large population of children with IF who were receiving care at a center for IR. Overall, compared with population norms, weight‐for‐age and length‐/height‐for‐age *z* scores were decreased at the time that PN was weaned, regardless of transplant status. Linear growth at the time of PN weaning was more delayed in those who received an intestinal transplant. In general, median weight gain and linear growth velocity remained stable throughout the 5‐year follow‐up period for children who did not receive an intestinal transplant. However, catch‐up growth occurred beginning at 2 years after PN weaning in those transplanted. After subdivision by primary diagnosis in children who did not receive an intestinal transplant, those with small bowel atresia faired best, experiencing growth acceleration beginning in year 3 after weaning. In those who were transplanted, children with NEC experienced catch‐up growth sooner than children with other diagnoses. In children who received a GLP‐2 analog, growth remained stable during treatment despite the population having a greater degree of growth delay at the time of medication start.

The demographics of our population appear to show a lower success rate regarding PN weaning than other cohorts previously reported. In our total population of children with IF, ~36% attained enteral autonomy. In children diagnosed with IF between 1986 and 2006, Modi et al reported that 67% achieved full enteral nutrition.^5^ Gattini et al reported a rate of enteral autonomy of 53% in a multicenter study of children diagnosed with IF between 2010 and 2015.^32^ Han et al reported a high rate of enteral autonomy (76%) in a population of adolescents (10–19 years) with SBS followed by a center for IR between 2009 and 2018,^37^ and Neelis et al reported that 71% of children with IF (2001–2015) who were receiving home PN were weaned in a median of 0.92 (IQR = 0.52–1.87) years.^19^ An earlier report by PIFCon described the natural history of pediatric IF in children diagnosed within the first year of age (*N* = 272) from 14 centers for IR between 2000 and 2004.^2^ After at least 2 years of follow‐up for all children, the cumulative incidence of enteral autonomy, defined as discontinuation of PN for >3 consecutive months, was 47%. It is important to note that some children in the current study were also participants in the PIFCon population. Two factors may account for the lower enteral autonomy rate that we observed. There are multiple definitions in the literature regarding the definition of IF and what constitutes enteral autonomy. Our definition is based on the American Society for Parenteral and Enteral Nutrition definition of enteral autonomy and is most consistent with the PIFCon paper. In addition, as a national referral center for intestinal transplant, there is a high proportion of patients with irreversible IF in ICARE awaiting or being evaluated for transplantation.

At the time that PN was discontinued in children who were not transplanted, 25% had a weight‐for‐age *z* score of <−2.0, and 33% had a length/height‐for‐age *z* score of <−2.0. The growth status of this subpopulation was like that observed by others who examined growth in children with IF who were receiving PN. Ali et al, while examining bone mineral status in children between ages 3 and 10 years who were subdivided into age groups, showed median *z* score values for weight‐for‐age, height‐for‐age, and BMI that ranged from −1.4 (IQR = −1.9 to −0.3) to −0.5 (IQR = −1.8 to 0.3), −1.3 (IQR = −2.6 to −0.6) to −1.8 (IQR = −2.4 to −1.3), and −0.3 (IQR = −1.1 to 0.6) to 0.8 (IQR = −0.2 to 1.1), respectively, where 25% of the population had a height‐for‐age *z* score of <−2.^38^ Olieman et al reported SD scores (SDSs) for weight and height of 0.1 ± 1.0 and −0.9 ± 1.3, respectively, which were significantly lower than reference values.^14^ Approximately half (53%) were below their midparental target height SDS.^14^ Pichler et al reported that 50% of children with SBS had a height SDS of <−2.0, which was defined as growth failure.^16^ In addition, the mean height was significantly lower in children with small bowel enteropathy compared with children diagnosed with a motility disorder (−2.5 ± 1.2 vs −1 ± 1.5, respectively; *P* = 0.007). The authors stated that children with enteropathies may be genetically predisposed to short stature^39^ and suggested that inflammation of the intestinal mucosa interferes with growth. Our population of children with small bowel enteropathy exhibited a similar degree of short stature to those in the Pichler et al study when PN was successfully weaned after transplantation. Growth velocity then accelerated in subsequent years.

Intestinal adaptation may take years in children, which may explain the lack of catch‐up growth observed in the majority of children who were not transplanted. We observed acceleration in linear growth in children with small bowel atresia and Hirschsprung disease, whereas post–PN wean growth was stable in children with the primary diagnosis of NEC. Deceleration in weight and length/height in children with midgut volvulus and gastroschisis began several years after weaning. Although the numbers are small when subdividing patients by primary diagnoses, these data warrant further investigation into whether the cause of IF could influence growth after PN weaning.

Our long‐term growth observations in children who did not receive an intestinal transplant are similar to an early study conducted by Goulet et al who examined growth over 15 years in children born between 1975 and 1991 who had an extensive neonatal small bowel resection.^17^ Those who were weaned from PN but later required parenteral or enteral support exhibited a significant decrease in weight and height within 4 years after PN cessation, whereas those who achieved enteral autonomy exhibited normal growth for at least 6 years after weaning. Roggero et al recently reported the growth pattern between birth and 24 months of age in infants with IF (*N* = 23) treated at a center for IR between 2015 and 2019.^10^ Although most infants had a birth weight and length that was appropriate for their gestational age, growth retardation developed during hospitalization. After discharge, weight and length *z* score values improved regardless of PN wean status. Neelis et al assessed growth in children with IF receiving home PN (*N* = 52) between 2001 and 2015.^19^ The majority of children (71%) weaned from PN (median duration of PN use = 0.92 years). Although weight gain was stable after PN weaning, a deceleration of linear growth velocity was observed (median duration of follow‐up = 3.35 years). Raphael et al previously described the natural history of growth in infants with SBS (*N* = 51) followed by a center for IR between 2003 and 2008.^8^ The median duration of PN was 4.8 years (IQR = 2.8–8.3). Plots of median gestational age–adjusted weight and length *z* scores revealed a U‐shaped curve with the highest values at birth (−0.28 and −0.41, respectively), the lowest values at 6 months (−2.38 and −2.18, respectively), and a rebound at 1 year of age (−0.72 and −0.76, respectively). A diagnosis of NEC was associated with lower weight *z* score and length *z* score in both univariate and multivariable regression analyses.

Achieving catch‐up growth after intestinal transplantation is partly dependent on the age of the child at the time of transplant, where velocity is expected to be greater in young children and during adolescence.^23^, ^40^ In our population, children who were weaned after transplant were toddlers. Our results are similar to those of earlier studies by Sudan et al, who reported normal or accelerated growth in 50% of children >1 year after transplant,^41^ and Ueno et al, who reported faster catch‐up growth in children >6 months posttransplant with pretransplant weight and length/height *z* scores of <−2.0.^42^ Venick et al reported steady increases in weight and length/height during the first year after transplant with stabilization in year 2,^24^ whereas children in our population experienced growth maintenance in year 1 and acceleration beginning in year 2. Another earlier study by Lacaille et al observed normal growth in two‐thirds of children followed into puberty.^43^ Moreover, of the six children who had completed growth, five achieved normal adult height (*z* score >−2.0). More recently, Courbage et al reported acceleration in linear growth in 64% of children who were >10 years after intestinal transplant.^25^ Acceleration in weight and linear growth in our transplant population continued through 5 years after PN weaning. Previous studies have not examined posttransplant growth by primary diagnosis. With the exception of two children with Hirschsprung disease who experienced a deceleration in linear growth, catch‐up growth was achieved in our transplant population regardless of diagnosis. Although results at transplant centers vary with regard to growth after pediatric intestinal transplantation, maintaining growth trajectory or achieving catch‐up growth with the possibility of reaching normal adult height appears to be possible for many children. Anthropometric measures should be monitored regularly to identify growth issues.^44^ Oral aversion and feeding difficulties are common complications in pediatric IF.^45^ Early identification of these problems is important so that interventions can be implemented while PN is being weaned.

The children who received teduglutide in our cohort had a median weight and length/height *z* score at the time of initiation that was much lower than the values reported in the ≥6‐month clinical trials designed to evaluate the safety and efficacy of teduglutide. The stable pattern of growth that we observed during teduglutide use was similar to previous research. However, we did observe an acceleration in weight gain during the first 6 months of medication therapy in ~40% of the cohort, which has not been reported previously. Kocoshis et al examined the parenteral support reduction rate after 24 weeks of GLP‐2 therapy in children with IF who received 0.025 mg/kg/day (*n* = 24), 0.05 mg/kg/day (*n* = 26), or standard care without GLP‐2 (*n* = 9).^29^ Parenteral support volume decreased by ≥20% during the intervention period in those who received GLP‐2 at the medium or high dose compared with those who received standard care (*P* < 0.05). There were no clinically significant changes in weight, height, or BMI *z* scores over the course of the intervention in either GLP‐2 group. Five children (10%) were weaned from parenteral support by the end of treatment. More recently, Lambe et al examined PN dependency and growth over 48 weeks of therapy with GLP‐2 (0.05 mg/kg/day) in children with SBS (*N* = 25) who had reached a plateau in intestinal adaptation.^30^ Compared with the baseline, the median number of infusions/week decreased from six to four and PN volume decreased. Growth trajectory was maintained throughout the study period. Eight children (32%) were weaned from parenteral support. Our rates of parenteral support weaning in children who received teduglutide (14% within 6 months and 36% within 12 months) were similar to the rates reported in the 24‐week clinical trial by Kocoshis et al and the 48‐week trial by Lambe et al.^29^, ^30^ These similarities occurred despite differences in our populations including degree of growth delay, unspecified indications for the use of a GLP‐2 analog, use of a GLP‐2 analog to reduce PN volume vs discontinuation of PN, and degree of aggressiveness with PN weaning.

We also sought to determine whether there were differences in the population based on the year of diagnosis owing to the evolution of the management of IF. The year 2013 was chosen based not only on the publication of the first multidisciplinary study defining the natural history of IF but also on the firm establishment of management principles, such as hepatic‐sparing PN and the prophylaxis/treatment of central line–associated bloodstream infections. Weight and length/height at the time of PN weaning did not differ statistically between children diagnosed with IF before or after 2013. Demographically, children diagnosed before 2013 displayed greater liver dysfunction, longer time receiving PN, and a reduced rate of PN weaning compared with children diagnosed more recently. Although this could represent a limitation in data interpretation, we decided to analyze the data in aggregate to assure adequate numbers.

This study has some limitations. Despite including children from two centers for IR and the large overall population size, the number of participants who weaned from PN in some of the diagnostic categories (Hirschsprung disease and microvillus inclusion disease) is small. The electronic medical record was initiated at UPMC Children's Hospital of Pittsburgh in the mid‐2000s. Study data for ICARE participants before the electronic medical record were available were obtained from paper registry documents where the information was collected in real time from paper medical records. Given the current inaccessibility of the paper records, we were unable to search for missing clinical information or confirm anthropometric values, if necessary. All participants were followed by a center for IR in which one of the primary goals is to provide adequate energy and macronutrients and micronutrients via parenteral and/or enteral support. However, the UPMC Children's Hospital of Pittsburgh is also a transplant center in which aggressiveness with PN weaning may be dictated by external factors, such as loss of venous access and recurrent life‐threatening infections. In addition, the ICARE population includes a high proportion of patients with irreversible IF who are on a transplant waiting list. We did not assess energy intake in those who were successfully weaned from PN or the percentage of energy from PN or enteral support or the PN dependency index (ratio of PN nonprotein energy intake/resting energy expenditure) in those who were receiving teduglutide to determine whether nutrition intake was adequate to support growth in our population.

## CONCLUSION

Children with IF exhibit growth delay when compared with growth reference standards. Given that PN is often discontinued before age 2 years and that intestinal adaptation can take months to years to occur, this population is at risk for significant growth delay. We observed long‐term stable weight gain and linear growth in a large population of children with IF who successfully weaned from PN in the absence of intestinal transplantation. Intestinal malabsorption may explain the observed maintenance of growth velocity vs catch‐up growth during the early years after PN weaning. The majority of children who received an intestinal transplant experienced early acceleration in weight and linear growth after PN weaning regardless of the primary diagnosis. Enteral intake should be assessed regularly after PN is discontinued in all children with IF to determine the adequacy of energy and macronutrients and micronutrient intake. Future studies to determine the enteral support requirements needed to achieve catch‐up growth are necessary. Anthropometric measures should also be monitored regularly to identify growth issues. Oral aversion and feeding difficulties are common complications in pediatric IF. Early identification of these problems is important so that interventions can be implemented while PN is being weaned. With the use of a GLP‐2 analog, weight *z* scores were maintained and even accelerated in the first 6 months of therapy. Length/height *z* score values revealed the maintenance of growth patterns over 3 years for most children receiving treatment. Longer follow‐up is required to determine the effect of pharmacologic treatment on growth in those who successfully wean from PN.

## AUTHOR CONTRIBUTIONS

Anita M. Nucci, Hillary Bashaw, Alexander Kirpich, and Jeffrey Rudolph contributed to the conception and design of the article. Anita M. Nucci contributed to the acquisition of the article material. Anita M. Nucci and Alexander Kirpich contributed to the analysis of the article data. Anita M. Nucci, Hillary Bashaw, and Jeffrey Rudolph contributed to the interpretation of the article material. Anita M. Nucci drafted the manuscript. All authors critically revised the article, agree to be fully accountable for ensuring the integrity and accuracy of the work, and for reading and approving the final article.

## CONFLICT OF INTEREST STATEMENT

The authors declare no conflict of interest.

## Supporting information

Figure S1. Weight, length/height, and body mass index *z* score values for children with intestinal failure who received teduglutide.

Supporting information.

Supporting information.
