# Supplementary figures and images for: Retrospective review of growth in pediatric intestinal failure after weaning from parenteral nutrition
Source: Nutr Clin Pract. 2024 Sep 12;40(1):176–87. doi: 10.1002/ncp.11209 (PMC11713205; doi:10.1002/ncp.11209)

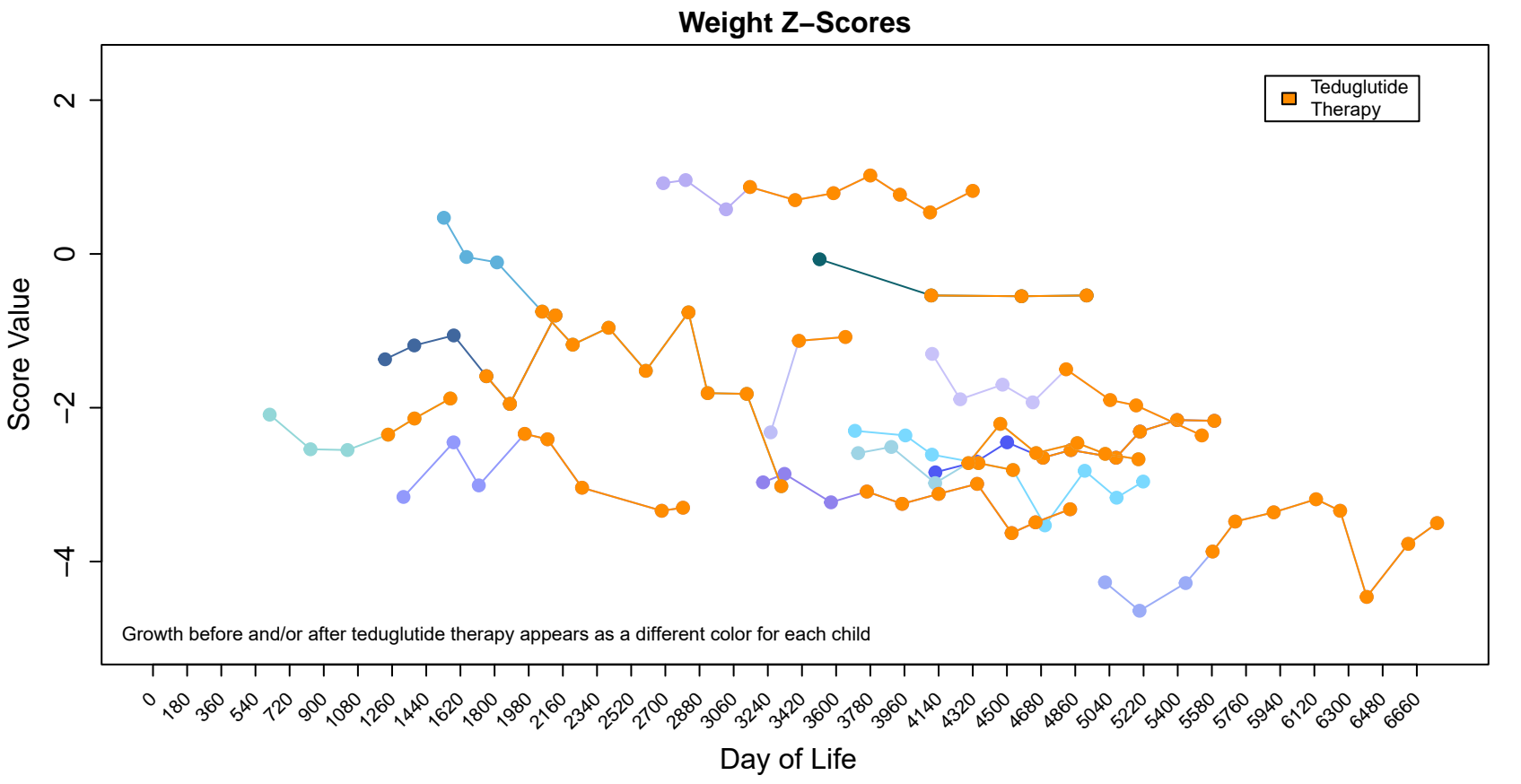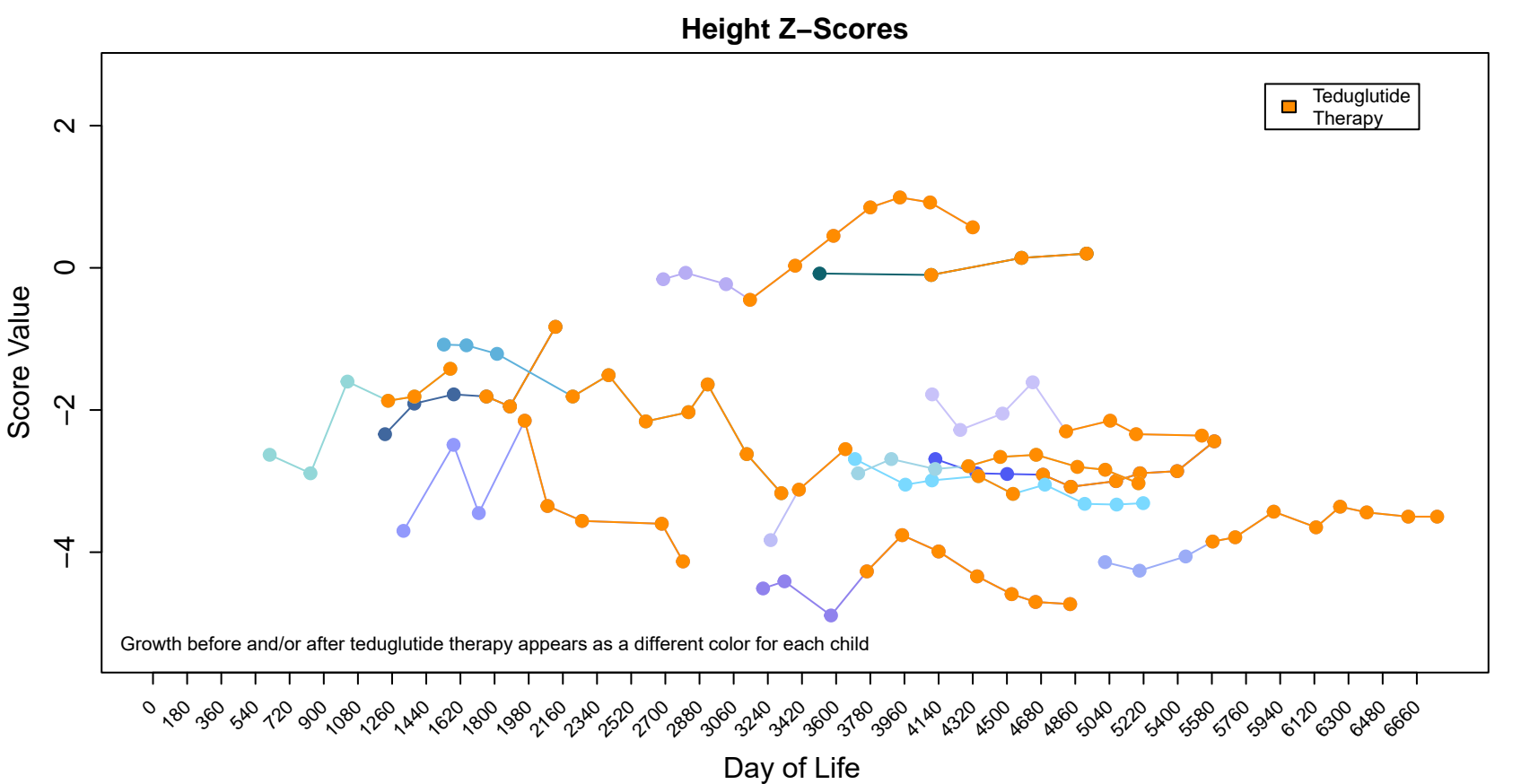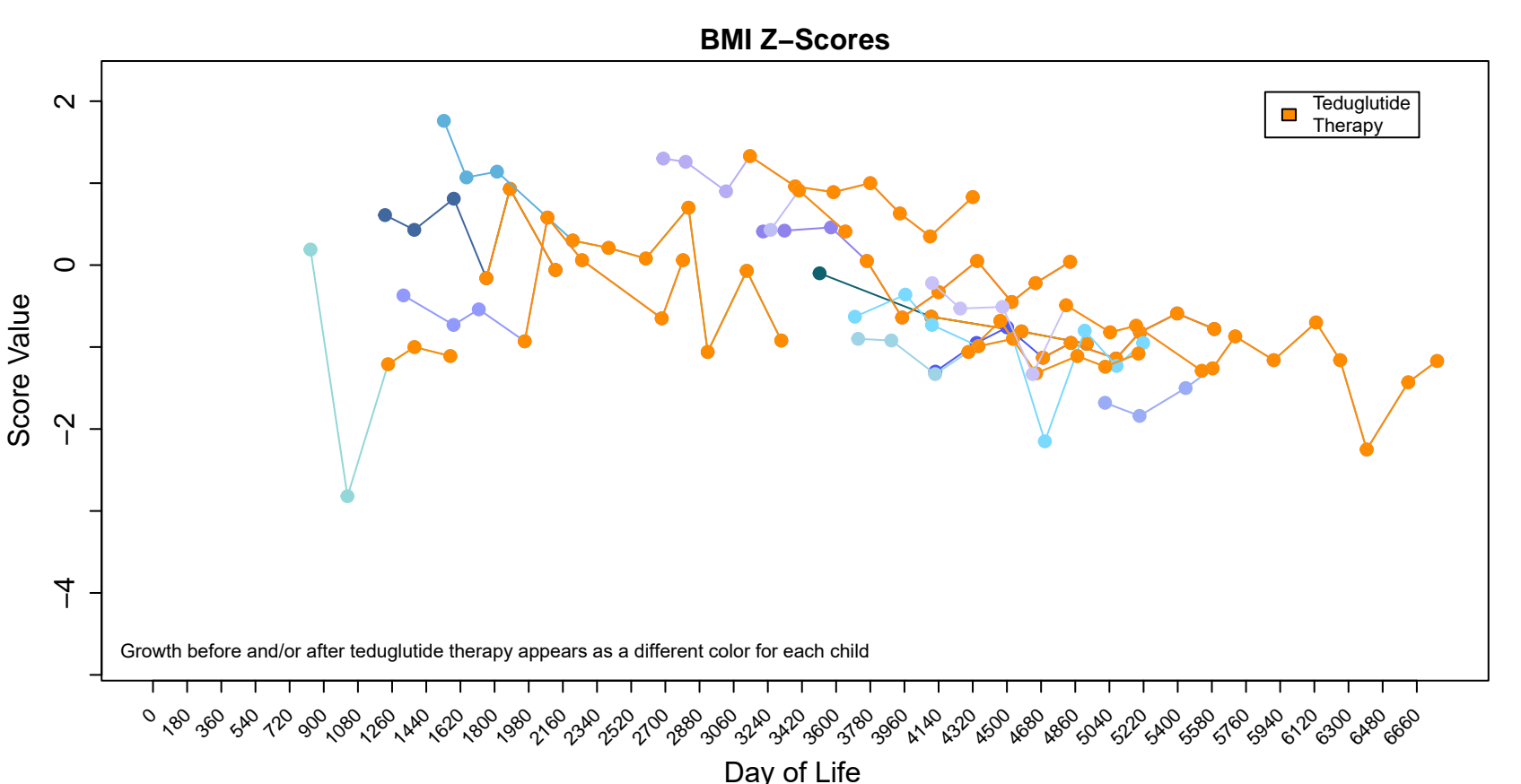

Supplement: Supplementary file 1 — Figure S1. Weight, length/height, and body mass index z score values for children with intestinal failure who received teduglutide. [file NCP-40-176-s001.pdf]
