# Supplementary material for: Retrospective review of growth in pediatric intestinal failure after weaning from parenteral nutrition
Source: Nutr Clin Pract. 2024 Sep 12;40(1):176–87. doi: 10.1002/ncp.11209 (PMC11713205; doi:10.1002/ncp.11209)
Supplement: Supplementary file 2 — Supporting information. [file NCP-40-176-s003.docx]

Table S1. Weight, Length/Height and Body Mass Index Z-Scores from Parenteral Nutrition Wean (Baseline) up to Five Years Post-Wean in Children with Intestinal Failure by Intestinal Transplant Status

| **Days** | **Baseline Weight** | **30** | **60** | **90** | **120** | **180** | **360** | **540** | **720** | **900** | **1080** | **1260** | **1440** | **1620** | **1800** |
| --- | --- | --- | --- | --- | --- | --- | --- | --- | --- | --- | --- | --- | --- | --- | --- |
| N | 144 | 144 | 143 | 143 | 141 | 138 | 129 | 122 | 114 | 107 | 99 | 95 | 92 | 90 | 86 |
| Total Population | -1.15  (-2.09, -0.32) | -1.19  (-2.09, -0.33) | -1.24  (-2.16, -0.41) | -1.28  (-2.15, -0.39) | -1.26  (-2.07, -0.32) | -1.32  (-2.15, -0.24) | -1.19  (-2.11, -0.31) | -1.2  (-2.19, -0.43) | -1.23  (-2.06, -0.28) | -1.33  (-2.06, -0.23) | -1.23  (-2.06, -0.29) | -1.23  (-2.08, -0.23) | -1.25  (-2.04, -0.34) | -1.29  (-2.25, -0.35) | -1.12  (-2.05, -0.4) |
|  | | | | | | | | | | | | | | | |
| n | 98 | 98 | 97 | 97 | 96 | 93 | 87 | 80 | 72 | 68 | 61 | 57 | 55 | 54 | 50 |
| No Intestinal Transplant | -1  (-2.08, -0.21) | -1  (-2.08, -0.21) | -1.09  (-2.11, -0.26) | -1.13  (-2.14, -0.15) | -1.07  (-2.04, -0.09) | -1.05  (-2.15, 0.09) | -1.06  (-2.3, -0.17) | -1.23  (-2.46, -0.38) | -1.23  (-2.22, -0.27) | -1.45  (-2.22, -0.33) | -1.26  (-2.35, -0.34) | -1.28  (-2.41, -0.34) | -1.5  (-2.24, -0.41) | -1.63  (-2.34, -0.45) | -1.65  (-2.48, -0.5) |
|  | | | | | | | | | | | | | | | |
| n | 46 | 46 | 46 | 46 | 45 | 45 | 42 | 42 | 42 | 39 | 38 | 38 | 37 | 36 | 36 |
| Intestinal Transplant | -1.27  (-2.08, -0.72) | -1.28  (-2.11, -0.85) | -1.37  (-2.26, -0.83) | -1.44  (-2.2, -0.81) | -1.53  (-2.14, -0.88) | -1.59  (-2.15, -0.91) | -1.38  (-1.98, -0.82) | -1.19  (-1.96, -0.54) | -1.2  (-1.93, -0.41) | -0.95  (-1.62, 0.24) | -0.93  (-1.8, 0.1) | -0.88  (-1.88, -0.1) | -0.67  (-1.75, -0.1) | -0.82  (-1.67, -0.18) | -0.77  (-1.64, -0.04) |

Z-scores expressed as median (Interquartile Range); Shading: No shading – stable growth (+0.5 z-score from baseline), green – acceleration (>+0.5 from baseline)

| **Days** | **Baseline Length/Height** | **30** | **60** | **90** | **120** | **180** | **360** | **540** | **720** | **900** | **1080** | **1260** | **1440** | **1620** | **1800** |
| --- | --- | --- | --- | --- | --- | --- | --- | --- | --- | --- | --- | --- | --- | --- | --- |
| N | 144 | 144 | 143 | 143 | 141 | 138 | 129 | 122 | 112 | 106 | 98 | 94 | 91 | 89 | 83 |
| Total Population | -1.89  (-2.9, -1.02) | -1.79  (-2.86, -0.99) | -1.77  (-2.85, -1.02) | -1.77  (-2.84, -0.79) | -1.79  (-2.88, -0.91) | -1.76  (-2.81, -0.98) | -1.72  (-2.86, -0.86) | -1.64  (-2.82, -0.84) | -1.67  (-2.9, -0.84) | -1.59  (-2.85, -0.82) | -1.46  (-2.8, -0.8) | -1.46  (-2.81, -0.69) | -1.45  (-2.81, -0.73) | -1.49  (-2.68, -0.69) | -1.51  (-2.67, -0.68) |
|  | | | | | | | | | | | | | | | |
| n | 98 | 98 | 97 | 97 | 96 | 93 | 87 | 80 | 71 | 67 | 60 | 56 | 54 | 53 | 47 |
| No Intestinal Transplant | -1.67  (-2.54, -0.72) | -1.6  (-2.56, -0.58) | -1.54  (-2.6, -0.49) | -1.44  (-2.61, -0.54) | -1.49  (-2.64, -0.58) | -1.66  (-2.7, -0.69) | -1.65  (-2.8, -0.71) | -1.58  (-2.81, -0.61) | -1.61  (-2.9, -0.71) | -1.62  (-2.79, -0.73) | -1.46  (-2.76, -0.83) | -1.52  (-2.77, -0.66) | -1.56  (-2.83, -0.75) | -1.73  (-2.75, -0.74) | -1.93  (-3.07, -0.68) |
|  | | | | | | | | | | | | | | | |
| n | 46 | 46 | 46 | 46 | 45 | 45 | 42 | 42 | 41 | 39 | 38 | 38 | 37 | 36 | 36 |
| Intestinal Transplant | -2.67  (-3.66, -1.77) | -2.55  (-3.45, -1.77) | -2.31  (-3.37, -1.56) | -2.43  (-3.33, -1.27) | -2.38  (-3.16, -1.35) | -2.53  (-2.93, -1.26) | -1.88  (-3.22, -1.24) | -1.68  (-2.93, -1.08) | -1.74  (-2.71 -1.01) | -1.46  (-2.98, -0.89) | -1.46  (-2.81, -0.74) | -1.34  (-2.62, -0.76) | -1.21  (-2.76, -0.67) | -0.99  (-2.65, -0.63) | -1.05  (-2.23, -0.63) |

Z-scores expressed as median (Interquartile Range); Shading: No shading – stable growth (+0.5 z-score from baseline), green – acceleration (>+0.5 from baseline)

| **Days** | **Baseline Body Mass Index** | **30** | **60** | **90** | **120** | **180** | **360** | **540** | **720** | **900** | **1080** | **1260** | **1440** | **1620** | **1800** |
| --- | --- | --- | --- | --- | --- | --- | --- | --- | --- | --- | --- | --- | --- | --- | --- |
| N | 45 | 45 | 45 | 45 | 45 | 43 | 42 | 40 | 40 | 37 | 33 | 31 | 30 | 28 | 27 |
| Total Population | 0.31  (-0.63, 0.83) | 0.35  (-0.96, 0.67) | 0.2  (-0.92, 0.7) | 0.17  (-0.88, 0.7) | 0.16  (-0.89, -.66) | 0.1  (-0.62, 0.6) | -0.14  (-0.89, -0.54) | -0.12  (-0.76, 0.55) | 0.07  (-0.78, 0.66) | -0.19  (-0.86, 0.35) | -0.37  (-0.83, 0.26) | -0.25  (-0.87, 0.23) | -0.18  (-1.09, 0.14) | -0.35  (-0.76, 0.13) | -0.43  (-0.84, 0.05) |
|  | | | | | | | | | | | | | | | |
| n | 33 | 33 | 33 | 33 | 33 | 31 | 30 | 28 | 28 | 26 | 23 | 21 | 21 | 20 | 19 |
| No Intestinal Transplant | 0.24  (-0.62, 0.83) | 0.23  (-0.7, 0.6) | 0.2  (-0.77, 0.73) | 0.17  (-0.84, 0.75) | 0.16  (-0.89, 0.77) | 0.1  (-0.54, 0.54) | -0.15  (-1.02, 0.5) | -0.12  (-0.89, 0.55) | 0.17  (-0.69, 0.67) | -0.16  (-0.85, 0.59) | -0.41  (-0.84, 0.41) | -0.29  (-1.01, 0.34) | -0.18  (-1.17, 0.23) | -0.35  (-0.76, 0.15) | -0.42  (-0.75, 0.05) |
|  | | | | | | | | | | | | | | | |
| n | 12 | 12 | 12 | 12 | 12 | 12 | 12 | 12 | 12 | 11 | 10 | 10 | 9 | 8 | 8 |
| Intestinal Transplant | 0.36  (-1.14, 0.89) | 0.4  (-1.1, 0.68) | 0.2  (-1.02, 0.64) | 0.09  (-0.94, 0.62) | 0.08  (-0.86, 0.63) | 0.06  (-0.71, 0.63) | 0.02  (-0.71, 0.69) | -0.12  (-0.67, 0.61) | -0.24  (-0.88, 0.44) | -0.19  (-0.86, 0.33) | -0.13  (-0.66, 0.13) | -0.18  (-0.43, 0.07) | -0.2  (-0.41, 0.09) | -0.36  (-0.74, 0.11) | -0.45  (-0.88, 0.05) |

Z-scores expressed as median (Interquartile Range); Shading: No shading – stable growth (+0.5 z-score from baseline), red – deceleration (>-0.5 from baseline)
