# Supplementary material for: Retrospective review of growth in pediatric intestinal failure after weaning from parenteral nutrition
Source: Nutr Clin Pract. 2024 Sep 12;40(1):176–87. doi: 10.1002/ncp.11209 (PMC11713205; doi:10.1002/ncp.11209)
Supplement: Supplementary file 3 — Supporting information. [file NCP-40-176-s002.pdf]

Table S2. Weight, Length/Height and Body Mass Index Z-Scores from Parenteral Nutrition Wean (Baseline) up to Five Years Post-Wean in Children with Intestinal Failure (Necrotizing Enterocolitis) by Intestinal Transplant Status

Days	Baseline Weight	30	60	90	120	180	360	540	720	900	1080	1260	1440	1620	1800
N	47	47	46	46	45	43	39	37	34	34	31	29	29	28	25
Total Necrotizing Enterocolitis	-1.36 (-2.23, -0.12)	-1.28 (-2.23, -0.19)	-1.24 (-2.28, -0.31)	-1.3 (-2.32, -0.31)	-1.47 (-2.24, -0.32)	-1.43 (-2.45, -0.31)	-1.62 (-2.4, -0.46)	-1.37 (-2.44, -0.45)	-1.01 (-2.19, -0.47)	-1.33 (-2.16, -0.45)	-1.25 (-2.24, -0.32)	-1.12 (-2.14, -0.3)	-1.14 (-2.04, -0.35)	-1.15 (-2.05, -0.19)	-1 (-1.99, -0.26)
n	42	42	41	41	41	39	35	33	30	30	27	25	25	24	21
No Intestinal Transplant	-1.41 (-2.29, -0.16)	-1.43 (-2.23, -0.23)	-1.65 (-2.24, -0.38)	-1.57 (-2.36, -0.39)	-1.53 (-2.24, -0.32)	-1.43 (-2.55, -0.31)	-1.52 (-2.4, -0.46)	-1.37 (-2.5, -0.45)	-1.34 (-2.21,-0.47)	-1.55 (-2.2, -0.65)	-1.25 (-2.3, -0.4)	-1.24 (-2.46, -0.57)	-1.28 (-2.04, -0.43)	-1.37 (-2.05, -0.26)	-1.09 (-1.99, -0.43)
n	5	5	5	5	4	4	4	4	4	4	4	4	4	4	4
Intestinal Transplant	-1.36 (-1.57, 0.29)	-1.03 (-1.28, 0.61)	-0.91 (-1.21, 0.92)	-0.87 (-1.32, 1.24)	-1.18 (-1.81, -0.33)	-1.34 (-1.93, -0.33)	-1.86 (-2.32, -0.82)	-1.55 (-2.02, -0.34)	-0.6 (-0.82, 0.01)	-0.26 (-1.04, 0.72)	-0.43 (-1.31, 0.66)	-0.37 (-1.31, 0.62)	-0.36 (-1.3, 0.55)	-0.36 (-1.4, 0.47)	-0.17 (-1.42, 0.69)

Z-scores expressed as Median (Interquartile range); Shading: No shading – stable growth (+0.5 z-score from baseline), green – acceleration (>+0.5 from baseline)

Days	Baseline Length/Height	30	60	90	120	180	360	540	720	900	1080	1260	1440	1620	1800
N	47	47	46	46	45	43	39	37	34	34	31	29	29	28	24
Total Necrotizing Enterocolitis	-1.78 (-2.8, -0.73)	-1.71 (-2.75, -0.58)	-1.73 (-2.71, -0.6)	-1.62 (-2.58, -0.65)	-1.7 (-2.68, -0.85)	-1.6 (-2.94, -0.87)	-1.81 (-2.88, -0.83)	-1.67 (-2.77, -0.78)	-1.75 (-2.89, -0.62)	-1.53 (-2.6, -0.49)	-1.42 (-2.59, -0.73)	-1.51 (-2.6, -0.49)	-1.51 (-2.46, -0.46)	-1.25 (-2.29, -0.5)	-1.28 (-2.11, -0.54)
n	42	42	41	41	41	39	35	33	30	30	27	25	25	24	20
No Intestinal Transplant	-1.72 (-2.76, -0.67)	-1.64 (-2.73, -0.5)	-1.72 (-2.72, -0.57)	-1.57 (-2.59, -0.65)	-1.42 (-2.6, -0.67)	-1.59 (-2.74, -0.87)	-1.78 (-2.79, -0.83)	-1.67 (-2.77, -0.78)	-1.61 (-2.89, -0.62)	-1.53 (-2.6, -0.49)	-1.42 (-2.59, -0.75)	-1.51 (-2.6, -0.56)	-1.53 (-2.46, -0.71)	-1.38 (-2.29, -0.64)	-1.45 (-2.11, -0.66)
n	5	5	5	5	4	4	4	4	4	4	4	4	4	4	4
Intestinal Transplant	-2.28 (-2.97, -2.02)	-2.1 (-2.89, -1.91)	-2.18 (-2.68, -1.54)	-2.41 (-2.51, -1.17)	-2.65 (-3.09, -2.19)	-3.04 (-3.38, -2.23)	-2.81 (-3.27, -1.66)	-2.12 (-2.92, -1.12)	-1.99 (-2.56, -1.27)	-1.59 (-2.3, -0.83)	-1.25 (-2.15, -0.36)	-0.95 (-2.21, 0.07)	-0. 81 (-2.07, 0.12)	-0.63 (-1.83, 0.09)	-0.63 (-1.89, 0.14)

Z-scores expressed as Median (Interquartile Range); Shading: No shading – stable growth (+0.5 z-score from baseline), green – acceleration (>+0.5 from baseline)

Days	Baseline Body Mass Index	30	60	90	120	180	360	540	720	900	1080	1260	1440	1620	1800
N	11	11	11	11	11	10	9	9	9	9	8	7	7	6	5
Total Necrotizing Enterocolitis	-0.23 (-1.1, 0.09)	-0.16 (-1.17, 0.1)	-0.22 (-1.44, 0.21)	-0.27 (-1.41, 0.22)	-0.32 (-1.29, 0.22)	-0.2 (-0.86, 0.25)	-0.29 (-1.06, 0.25)	-0.07 (-0.94, 0.24)	-0.12 (-1.02, 0.56)	-0.62 (-0.81, 0.25)	-0.31 (-0.54, -0.04)	-0.25 (-0.87, 0.31)	-0.12 (-0.89, 0.13)	0.02 (-0.39, 0.12)	-0.11 (-0.17, 0.57)
n	11	11	11	11	11	10	9	9	9	9	8	7	7	6	5
No Intestinal Transplant	-0.23 (-1.1, 0.09)	-0.16 (-1.17, 0.1)	-0.22 (-1.44, 0.21)	-0.27 (-1.41, 0.22)	-0.32 (-1.29, 0.22)	-0.2 (-0.86, 0.25)	-0.29 (-1.06, 0.25)	-0.07 (-0.94, 0.24)	-0.12 (-1.02, 0.56)	-0.62 (-0.81, 0.25)	-0.31 (-0.54, -0.04)	-0.25 (-0.87, 0.31)	-0.12 (-0.89, 0.13)	0.02 (-0.39, 0.12)	-0.11 (-0.17, 0.57)
n	0	0	0	0	0	0	0	0	0	0	0	0	0	0	0
Intestinal Transplant	--	--	--	--	--	--	--	--	--	--	--	--	--	--	--

Z-scores expressed as Median (Interquartile Range); Shading: No shading – stable growth (+0.5 z-score from baseline), green – acceleration (>+0.5 from baseline)

Table S3. Weight, Length/Height and Body Mass Index Z-Scores from Parenteral Nutrition Wean (Baseline) up to Five Years Post-Wean in Children with Intestinal Failure (Gastroschisis) by Intestinal Transplant Status

Days	Baseline Weight	30	60	90	120	180	360	540	720	900	1080	1260	1440	1620	1800
N	23	23	23	23	23	23	22	21	20	16	16	16	16	16	16
Total Gastroschisis	-1.26 (-1.91, -0.29)	-1.28 (-1.93, -1.28)	-1.29 (-1.93, -0.36)	-1.3 (-1.92, -0.4)	-1.34 (-1.86, -0.44)	-1.39 (-1.7, -0.54)	-1.25 (-2.07, -0.8)	-1.19 (-1.9, -0.59)	-1.21 (-1.91, -0.52)	-1.33 (-1.7, -0.4)	-1.36 (-1.8, -0.17)	-1.34 (-1.86, -0.25)	-1.46 (-1.83, -0.63)	-1.52 (-1.98, -0.76)	-1.5 (-2.14, -0.55)
n	12	12	12	12	12	12	12	11	10	7	7	7	7	7	7
No Intestinal Transplant	-0.83 (-1.16, 0.29)	-0.91 (-1.2, 0.35)	-0.93 (-1.26, 0.35)	-0.93 (-1.34, -0.08)	-0.93 (-1.44, -0.09)	-0.91 (-1.62, -0.2)	-0.84 (-1.74, -0.44)	-1.19 (-1.64, -0.8)	-1.06 (-1.68, -0.64)	-1.55 (-1.7, -0.92)	-1.38 (-1.59, -1.11)	-1.43 (-1.72, -1.17)	-1.58 (-1.93, -1.33)	-1.97 (-2.13, -1.49)	-2.07 (-2.43, -1.53)
n	11	11	11	11	11	11	10	10	10	9	9	9	9	9	9
Intestinal Transplant	-1.64 (-2.27, -1.27)	-1.66 (-2.29, -1.32)	-1.67 (-2.28, -1.36)	-1.66 (-2.22, -1.39)	-1.57 (-2.09, 1.39)	-1.59 (-1.85, -1.36)	-1.45 (-2.07, -1.22)	-1.21 (-1.96, -0.64)	-1.49 (-1.95, -0.17)	-0.94 (-1.65, 0.19)	-0.73 (-1.94, 0.18)	-0.69 (-2.06, -0.24)	-0.67 (-1.75, -0.53)	-0.92 (-1.65, -0.72)	-0.95 (-1.61, -0.4)

Z-scores expressed as median (Interquartile Range); Shading: No shading – stable growth (+0.5 z-score from baseline), green – acceleration (>+0.5 from baseline), red – deceleration (>-0.5 from baseline)

Days	Baseline Length/Height	30	60	90	120	180	360	540	720	900	1080	1260	1440	1620	1800
N	23	23	23	23	23	23	22	21	20	16	16	16	16	16	16
Total Gastroschisis	-1.8 (-2.95, -0.88)	-1.78 (-3.16, -0.78)	-1.78 (-3.38, -0.9)	-1.77 (-3.32, -0.76)	-1.76 (-3.17, -0.54)	-1.69 (-2.81, -0.59)	-1.69 (-2.81, -0.72)	-1.86 (-2.56, -0.82)	-1.87 (-2.76, -1.21)	-2.05 (-2.9, -0.96)	-1.94 (-2.29, -0.64)	-1.76 (-2.32, -0.65)	-1.65 (-2.31, -0.77)	-1.68 (-2.48, -0.86)	-1.75 (-2.71, -0.73)
n	12	12	12	12	12	12	12	11	10	7	7	7	7	7	7
No Intestinal Transplant	-1.39 (-1.89, -0.68)	-1.64 (-1.89, -0.48)	-1.53 (-1.85, -0.28)	-1.41 (-1.94, -0.17)	-1.5 (-1.85, 0.05)	-1.53 (-1.89, -0.07)	-1.53 (-2.36, 0.02)	-1.73 (-2.55, -0.51)	-2.12 (-2.76, -1.14)	-2.73 (-3.14, -1.04)	-2.18 (-2.9, -1.02)	-2.23 (-2.93, -1.1)	-2.25 (-2.76, -1.21)	-2.46 (-2.8, -1.31)	-2.61 (-3.1, -1.39)
n	11	11	11	11	11	11	10	10	10	9	9	9	9	9	9
Intestinal Transplant	-2.73 (-3.79, -1.57)	-3.09 (-3.68, -1.51)	-3.28 (-3.52, -1.44)	-3.3 (-3.36, -1.38)	-3.16 (-3.25, -1.34)	-2.77 (-2.9, -1.27)	-2.08 (-3.23, -1.2)	-1.9 (-2.83, -1.39)	-1.82 (-2.35, -1.39(	-1.74 (-2.35, -1.01)	-1.72 (-2.22, -0.72)	-1.58 (-1.86, -0.65)	-1.21 (-1.8, -0.83)	-1.09 (-1.94, -0.95)	-1.18 (-2.08, -0.76)

Z-scores expressed as median (Interquartile Range); Shading: No shading – stable growth (+0.5 z-score from baseline), green – acceleration (>+0.5 from baseline), red – deceleration (>-0.5 from baseline)

Days	Baseline Body Mass Index	30	60	90	120	180	360	540	720	900	1080	1260	1440	1620	1800
N	7	7	7	7	7	7	7	7	7	5	5	5	5	5	5
Total Gastroschisis	0.54 (0.39, 1.02)	0.53 (0.44, 1.07)	0.73 (0.43, 1.09)	0.92 (0.43, 1.13)	0.89 (0.42, 1.15)	0.44 (0.34, 0.84)	-0.01 (-0.43, 0.85)	0.1 (-0.18, 0.66)	0.75 (0.24, 0.87)	0.35 (-0.01, 1)	0.14 (-0.03, 0.87)	-0.01 (-0.04, 0.34)	-0.15 (-0.18, -0.11)	-0.3 (-0.32, -0.25)	-0.45 (-0.56, -0.4)
n	6	6	6	6	6	6	6	6	6	4	4	4	4	4	4
No Intestinal Transplant	0.49 (0.36, 0.78)	0.53 (0.4, 0.84)	0.62 (0.39, 0.95)	0.71 (0.39, 1.05)	0.69 (0.39, 1.06)	0.41 (0.33, 0.45)	-0.03 (-0.62, 0.28)	-0.04 (-0.19, 0.14)	0.59 (0.14, 0.88)	0.5 (-0.22, 1.17)	0.42 (-0.17, 0.89)	0.15 (-0.1, 0.41)	-0.14 (-0.18, 0.04)	-0.29 (-0.33, -0.07)	-0.48 (-0.57, -0.16)
n	1	1	1	1	1	1	1	1	1	1	1	1	1	1	1
Intestinal Transplant*	1.26	1.21	1.16	1.15	1.17	1.22	1.34	1.29	0.82	0.35	0.14	-0.01	-0.15	-0.3	-0.45

Z-scores expressed as median (Interquartile Range); Shading: No shading – stable growth (+0.5 z-score from baseline), green – acceleration (>+0.5 from baseline), red – deceleration (>-0.5 from baseline)

*Median only

Table S4. Weight, Length/Height and Body Mass Index Z-Scores from Parenteral Nutrition Wean (Baseline) up to Five Years Post-Wean in Children with Intestinal Failure (Small Bowel Atresia) by Intestinal Transplant Status

Days	Baseline Weight	30	60	90	120	180	360	540	720	900	1080	1260	1440	1620	1800
N	24	24	24	24	23	23	22	21	17	16	13	12	11	11	11
Total Small Bowel Atresia	-0.91 (-1.78, -0.54)	-1.02 (-1.92, -0.27)	-1.2 (-2.06, -0.34)	-1.31 (-2.03, -0.14)	-1.08 (-1.86, -0.01)	-0.9 (-1.82, -0.06)	-0.57 (-1.39, -0.14)	-0.54 (-1.31, -0.13)	-0.47 (-1.39, -0.09)	-0.44 (-1.43, 0.06)	-0.4 (-1.15, 0.12)	-0.57 (-0.92, 0.07)	-0.52 (-1.16, -0.11)	-0.44 (-1.89, -0.06)	-0.49 (-1.72, -0.18)
n	18	18	18	18	17	17	16	15	11	11	9	8	7	7	7
No Intestinal Transplant	-1.19 (-1.91, -0.33)	-1.24 (-1.94, -0.21)	-1.31 (-2, -0.13)	-1.31 (-1.98, -0.09)	-1.08 (-1.79, 0.08)	-0.87 (-1.19, 0.09)	-0.41 (-1.45, -0.17)	-0.52 (-1.19, -0.21)	-0.27 (-1.47, -0.15)	-0.34 (-1.64, -0.13)	-0.4 (-1.15, -.12)	-0.57 (-1.05, 0.07)	-0.74 (-1.4, -0.3)	-0.73 (-2.29, -0.34)	-0.62 (-1.84, -0.44)
n	6	6	6	6	6	6	6	6	6	5	4	4	4	4	4
Intestinal Transplant	-0.81 (-1.03, -0.63)	-0.92 (-1.72, -0.63)	-1.01 (-1.91, -0.58)	-1.09 (-1.92, -0.53)	-1.18 (-1.94, -0.48)	-1.35 (-1.93, -0.37)	-1.04 (-1.34, 0.11)	-0.79 (-1.24, 0.43)	-0.59 (-1.22, 0.49)	-0.54 (-1.27, 0.51)	-0.74 (-1.13, -0.03)	-0.51 (-0.92, 0.11)	-0.27 (-0.74, 0.24)	-0.04 (-0.55, 0.38)	0.09 (-0.52, 0.49)

Z-scores expressed as median (Interquartile Range); Shading: No shading – stable growth (+0.5 z-score from baseline), green – acceleration (>+0.5 from baseline)

Days	Baseline Length/Height	30	60	90	120	180	360	540	720	900	1080	1260	1440	1620	1800
N	24	24	24	24	23	23	22	21	16	16	13	12	11	11	10
Total Small Bowel Atresia	-1.53 (-2, -0.75)	-1.59 (-1.98, -0.88)	-1.53 (-2.13, -0.96)	-1.55 (-2, -0.92)	-1.72 (-2.04, -1.29)	-1.86 (-2.21, -1.09)	-1.59 (-2.06, -0.88)	-1.28 (-1.67, -0.86)	-1.32 (-1.47, -0.88)	-1.16 (-1.36, -0.75)	-0.98 (-1.17, -0.8)	-0.89 (-1.12, -0.53)	-0.95 (-1.15, -0.76)	-1.12 (-1.39, -0.8)	-0.97 (-1.47, -0.75)
n	18	18	18	18	17	17	16	15	11	11	9	8	7	7	6
No Intestinal Transplant	-1.53 (-1.97, -0.83)	-1.57 (-1.78, -0.8)	-1.48 (-1.88, -0.77)	-1.4 (-1.92, -0.72)	-1.55 (-2.04, -1.07)	-1.86 (-2.15, -1.1)	-1.57 (-2.05, -0.59)	-1.2 (-1.64, -0.47)	-1.35 (-1.5, -1.01)	-1.19 (-1.43, -0.94)	-0.98 (-1.06, -0.83)	-0.89 (-1.12, -0.47)	-1.12 (-1.15, -0.85)	-1.15 (-1.39, -1.02)	-1.15 (-1.47, -0.93)
n	6	6	6	6	6	6	6	6	5	5	4	4	4	4	4
Intestinal Transplant	-1.47 (-2.86, -0.48)	-1.77 (-2.52, -1.27)	-1.92 (-2.24, -1.25)	-1.79 (-1.99, -1.22)	-1.56 (-1.93, -1.22)	-1.39 (-1.84, -0.99)	-1.07 (-1.5, -0.77)	-1.18 (-1.45, -0.82)	-0.9 (-1.38, -0.8)	-0.81 (-1.27, -0.58)	-0.98 (-1.32, -0.66)	-0.92 (-1.23, -0.57)	-0.88 (-1.17, -0.48)	-0.8 (-1.1, -0.4)	-0.77 (-1.08, -0.39(

Z-scores expressed as median (Interquartile Range); Shading: No shading – stable growth (+0.5 z-score from baseline), green – acceleration (>+0.5 from baseline)

Days	Baseline Body Mass Index	30	60	90	120	180	360	540	720	900	1080	1260	1440	1620	1800
N	4	4	4	4	4	4	4	4	4	4	3	3	3	3	3
Total Small Bowel Atresia	-0.78 (-0.95, -0.39)	-0.83 (-0.97, -0.42)	-0.85 (-0.95, -0.45)	-0.86 (-0.94, -0.49)	-0.86 (-0.96, -0.46)	-0.83 (-1.02, -0.35)	-0.91 (-1.09, -0.15)	-1.02 (-1.29, -0.15)	-1.1 (-1.42, -0.28)	-1.06 (-1.49, -0.29)	-1.6 (-1.7, -1)	-1.63 (-1.77, -0.89)	-1.68 (-2.25, -0.8)	-2.11 (-2.42, -0.89)	-1.85 (-2.24, -0.71)
n	2	2	2	2	2	2	2	2	2	2	2	2	2	2	2
No Intestinal Transplant	-0.78 (-0.85, -0.7)	-0.85 (-0.92, -0.77)	-0.91 (-0.98, -0.84)	-0.98 (-1.05, -0.91)	-1.04 (-1.11, -1.02)	-1.11 (-1.21, -1.02)	-1.1 (-1.11, -1.09)	-1.31 (-1.33, -1.29)	-1.43 (-1.44, -1.42)	-1.51 (-1.52, -1.49)	-1.7 (-1.75, -1.65)	-1.77 (-1.84, -1.65)	-2.25 (-2.53, -1.97)	-2.42 (-2.58, -2.26)	-2.24 (-2.43, -2.05)
n	2	2	2	2	2	2	2	2	2	2	1	1	1	1	1
Intestinal Transplant*	-0.35 (-0.68, -0.02)	-0.28 (-0.62, 0.06)	-0.22 (-0.57, 0.14)	-0.15 (-0.51, 0.21)	-0.08 (-0.46, 0.29)	0.05 (-0.35, 0.44)	0.44 (-0.15, 1.02)	0.46 (-0.15, 1.06)	0.22 (-0.28, 0.72)	0.06 (-0.29, 0.41)	-0.4	-0.15	0.09	0.34	0.44

Z-scores expressed as median (Interquartile Range); Shading: No shading – stable growth (+0.5 z-score from baseline), green – acceleration (>+0.5 from baseline), red – deceleration (>-0.5 from baseline); *Median only when n=1

Table S5. Weight, Length/Height and Body Mass Index Z-Scores from Parenteral Nutrition Wean (Baseline) up to Five Years Post-Wean in Children with Intestinal Failure (Midgut Volvulus) by Intestinal Transplant Status

Days	Baseline Weight	30	60	90	120	180	360	540	720	900	1080	1260	1440	1620	1800
N	20	20	20	20	20	20	19	18	18	18	17	17	17	17	17
Total Midgut Volvulus	-1.33<br>(-2.27, -0.6)	-1.23<br>(-2.39, -0.7)	-1.21<br>(-2.81, -0.48)	-1.29<br>(-2.64, -0.5)	-1.47<br>(-2.52, -0.58)	-1.55<br>(-2.2, -0.52)	-1.09<br>(-1.85, -0.51)	-1.28<br>(-2.51, -0.28)	-1.4<br>(-2.14, -0.43)	-1.15<br>(-1.94, -0.36)	-1.37<br>(-2.35, -0.31)	-1.43<br>(-2.43, -0.21)	-1.43<br>(-2.75, -0.27)	-1.41<br>(-3.05, -0.45)	-1.48<br>(-3.36, -0.57)
n	10	10	10	10	10	10	9	8	8	8	7	7	7	7	7
No Intestinal Transplant	-0.76<br>(-1.84, -0.28)	-0.76<br>(-1.91, -0.36)	-0.76<br>(-2, -0.36)	-0.77<br>(-2.17, -0.05)	-0.83<br>(-2.35, 0.3)	-0.93<br>(-2.28, 0.45)	--1.09<br>(-2.54, -0.35)	-1.47<br>(-2.95, -0.39)	-1.4<br>(-3.17, -0.46)	-1.64<br>(-3.32, -0.52)	-2.35<br>(-3.79, -1.22)	-1.61<br>(-4.06, -1.36)	-1.6<br>(-4.29, -1.5)	-1.73<br>(-4.46, -1.4)	-1.86<br>(-4.34, -1.47)
n	10	10	10	10	10	10	10	10	10	10	10	10	10	10	10
Intestinal Transplant	-1.54<br>(-2.44, -1.01)	-1.67<br>(-2.59, -0.9)	-1.47<br>(-2.83, -1.09)	-1.51<br>(-2.68, -0.96)	-1.65<br>(-2.5, -1.03)	-1.79<br>(-2.13, -1.09)	-1.25<br>(-1.66, -0.67)	-1.03<br>(-1.94, -0.28)	-1.46<br>(-2.03, -0.43)	-0.92<br>(-1.72, -0.36)	-0.68<br>(-1.8, -0.18)	-0.73<br>(-1.75, -0.11)	-0.8<br>(-1.7, -0.14)	-0.84<br>(-1.64, -0.36)	-0.8<br>(-1.58, -0.37)

Z-scores expressed as median (Interquartile Range); Shading: No shading – stable growth (+0.5 z-score from baseline), green – acceleration (>+0.5 from baseline), red – deceleration (>-0.5 from baseline)

Days	Baseline Length/Height	30	60	90	120	180	360	540	720	900	1080	1260	1440	1620	1800
N	20	20	20	20	20	20	19	18	18	18	17	17	17	17	17
Total Midgut Volvulus	-2.9<br>(-3.75, -1.66)	-2.77<br>(-3.49, -1.34)	-2.71<br>(-3.32, -1.31)	-2.69<br>(-3.23, -1.28)	-2.73<br>(-3.19, -1.33)	-2.6<br>(-3.31, -1.14)	-2.11<br>(-3.35, -1.48)	-2.28<br>(-3.47, -1.28)	-2.15<br>(-3.4, -1.28)	-2.2<br>(-3.22, -0.94)	-2.41<br>(-3.34, -0.94)	-2.29<br>(-3.56, -0.86)	-2.3<br>(-3.78, -1)	-2.02<br>(-4, -0.98)	-2.1<br>(-4.06, -0.98)
n	10	10	10	10	10	10	9	8	8	8	7	7	7	7	7
No Intestinal Transplant	-1.94<br>(-2.86, -0.43)	-1.77<br>(-2.88, -0.38)	-1.82<br>-2.91, -0.33)	-2.07<br>(-2.98, -0.5)	-2.17<br>(-3.28, -0.62)	-2.33<br>(-3.36, -0.8)	-2.95<br>(-3.36, -1.24)	-2.85<br>(-3.46, -1.22)	-2.54<br>(-3.42, -1.66)	-2.68<br>(-3.32, -1.02)	-3.26<br>(-3.89, -1.68)	-3.27<br>(-3.98, -1.57)	-3.24<br>(-4.23, -1.67)	-3.2<br>(-4.38, -1.99)	-3.16<br>(-4.48, -2.05)
n	10	10	10	10	10	10	10	10	10	10	10	10	10	10	10
Intestinal Transplant	-2.91<br>(-3.59, -2.91)	-3.22<br>(-3.79, -2.56)	-3.03<br>(-3.54, -2.2)	-2.81<br>(-3.33, -2.25)	-2.77<br>(-3.14, -2.1)	-2.6<br>(-3.1, -1.83)	-1.95<br>(-3.09, -1.63)	-1.92<br>(-3.23, -1.34)	-2<br>(-3.23, -1.28)	-2<br>(-3.22, -0.92)	-1.96<br>(-3.2, -0.75)	-1.96<br>(-3.17, -0.88)	-1.73<br>(-3.04, -0.83)	-1.5<br>(-2.86, -0.77)	-1.51<br>(-2.69, -0.78)

Z-scores expressed as median (Interquartile Range); Shading: No shading – stable growth (±0.5 z-score from baseline), green – acceleration (>+0.5 from baseline), red – deceleration (>-0.5 from baseline)

Days	Baseline Body Mass Index	30	60	90	120	180	360	540	720	900	1080	1260	1440	1620	1800
N	6	6	6	6	6	6	6	5	5	5	4	4	4	4	4
Total Midgut Volvulus	0.68<br>(0.44, 0.9)	0.56<br>(0.42, 0.77)	0.52<br>(0.23, 0.78)	0.55<br>(0.09, 0.79)	0.55<br>(-0.05, 0.79)	0.5<br>(-0.33, 0.77)	0.53<br>(0.39, 0.7)	0.45<br>(0.4, 0.7)	0.24<br>(0.22, 0.47)	0.35<br>(-0.21, 0.58)	-0.1<br>(-0.6, 0.39)	0.29<br>(-0.1, 0.35)	-.21<br>(-0.18, 0.24)	0.14<br>(-0.02, 0.17)	0.03<br>(-0.11, 0.06)
n	5	5	5	5	5	5	5	4	4	4	3	3	3	3	3
No Intestinal Transplant	0.83<br>(0.52, 0.92)	0.6<br>(0.52, 0.82)	0.67<br>(0.19, 0.82)	0.75<br>(0.01, 0.81)	0.77<br>(-0.17, 0.8)	0.71<br>(-0.54, 0.79)	0.54<br>(0.35, 0.75)	0.55<br>(0, 0.73)	0.35<br>(0.21, 0.51)	0.18<br>(-0.22, 0.58)	-0.57<br>(-0.63, -0.04)	0.25<br>(-0.45, 0.32)	0.23<br>(-0.53, 0.26)	0.16<br>(-0.15, 0.18)	0<br>(-0.22, 0.05)
n	1	1	1	1	1	1	1	1	1	1	1	1	1	1	1
Intestinal Transplant*	0.41	0.39	0.37	0.35	0.33	0.29	0.52	0.45	0.22	0.35	0.36	0.34	0.19	0.12	0.05

Z-scores expressed as median (Interquartile Range); No shading – stable growth (+0.5 z-score from baseline), red – deceleration (>-0.5 from baseline); *Median only

Table S6. Weight Length/Height and Body Mass Index Z-Scores from Parenteral Nutrition Wean (Baseline) up to Five Years Post-Wean in Children with Intestinal Failure (Hirschsprung's Disease) by Intestinal Transplant Status

Days	Baseline Weight	30	60	90	120	180	360	540	720	900	1080	1260	1440	1620	1800
N	4	4	4	4	4	4	4	4	4	4	4	4	3	3	3
Total Hirschsprung's Disease	-1.21 (-1.46, -0.55)	-1.21 (-1.34, -0.55)	-1.19 (-1.27, -0.54)	-1.05 (-1.15, -0.47)	-0.9 (-1.13, -0.31)	-0.56 (-1.07, 0.07)	0.03 (-0.58, 0.39)	-0.55 (-0.97, -0.34)	-0.54 (-0.91, -0.34)	-0.17 (-0.82, 0.14)	-0.2 (-0.73, 0.14)	-0.31 (-0.79, -0.07)	-0.35 (-1.11, -0.04)	-0.46 (-1.15, 0.03)	-0.57 (-1.18, 0.1)
n	2	2	2	2	2	2	2	2	2	2	2	2	1	1	1
No Intestinal Transplant*	-0.38 (-1.28, 0.51)	-0.13 (-0.86, 0.59)	-0.01 (-0.64, 0.61)	0.07 (-0.47, 0.61)	0.15 (-0.31, 0.61)	0.35 (0.07, 0.62)	0.39 (0.11, 0.66)	-0.14 (-0.34,-0.05)	-0.14 (-0.34, 0.05)	-0.17 (-0.3, -0.04)	-0.2 (-0.26, -0.13)	-0.31 (-0.37, -0.26)	-0.35	-0.46	-0.57
n	2	2	2	2	2	2	2	2	2	2	2	2	2	2	2
Intestinal Transplant	-1.21 (-1.22, -1.21)	-1.21 (-1.23, -1.2)	-1.22 (-1.26, -1.17)	-1.22 (-1.29, -1.15)	-1.23 (-1.32, 1.13)	-1.22 (-1.37, -1.07)	-0.82 (-1.34, -0.3)	-1.37 (-1.77, -0.97)	-1.29 (-1.66, -0.91)	-0.84 (-1.42, -0.26)	-0.59 (-1.28, 0.09)	-0.78 (-1.35, -0.21)	-0.81 (-1.34, -0.27)	-0.66 (-1.25, -0.06)	-0.51 (-1.16, 0.13)

Z-scores expressed as median (Interquartile Range); Shading: No shading – stable growth (+0.5 z-score from baseline), green – acceleration (>+0.5 from baseline), red – deceleration (>-0.5 from baseline); *Median only when n=1

Days	Baseline Length/Height	30	60	90	120	180	360	540	720	900	1080	1260	1440	1620	1800
N	4	4	4	4	4	4	4	4	4	4	4	4	3	3	3
Total Hirschsprung's Disease	-1.66 (-2.13, -0.85)	-1.29 (-1.73, -0.68)	-1.08 (-1.61, -0.52)	-0.96 (-1.6, -0.35)	-0.83 (-1.6, -0.18)	-0.51 (-1.42, 0.09)	-1.18 (-1.67, -0.71)	-0.95 (-1.63, -0.61)	-0.77 (-1.58, -0.4)	-0.51 (-1.31, -0.18)	-0.29 (-1.35, 0.28)	-0.12 (-1.2, 0.37)	0.29 (-1.32, 0.35)	-0.07 (-1.45, 0.17)	-0.56 (-1.64, -0.06)
n	2	2	2	2	2	2	2	2	2	2	2	2	1	1	1
No Intestinal Transplant*	-0.98 (-1.6, -0.36)	-0.67 (-1.13, -0.21)	-0.57 (-0.99, -0.16)	-0.56 (-0.96, -0.16)	-0.55 (-0.94, -0.15)	-0.41 (-0.73, -0.09)	-0.31 (-0.71, 0.08)	-0.39 (-0.61, -0.16)	-0.26 (-0.4, -0.12)	-0.2 (-0.44, 0.04)	-0.12 (-0.44, 0.21)	-0.08 (-0.34, 0.17)	0.29	-0.07	-0.56
n	2	2	2	2	2	2	2	2	2	2	2	2	2	2	2
Intestinal Transplant	-1.66 (-1.88, -1.44)	-1.58 (-1.88, -1.29)	-1.51 (-1.87, -1.14)	-1.43 (-1.87, -0.99)	-1.35 (-1.87, -0.84)	-1.24 (-1.89, -0.6)	-2.1 (-2.52, -1.67)	-2.21 (-2.8, -1.63)	-2.16 (-2.73, -1.58)	-1.77 (-2.49, -1.05)	-1.46 (-2.29, -0.63)	-1.34 (-2.18, -0.49)	-1.26 (-2.09, -0.43)	-1.21 (-2.02, -0.4)	-1.15 (-1.94, -0.36)

Z-scores expressed as median (Interquartile Range); Shading: No shading – stable growth (+0.5 z-score from baseline), green – acceleration (>+0.5 from baseline), red – deceleration (>-0.5 from baseline); *Median only when n=1

Days	Baseline Body Mass Index	30	60	90	120	180	360	540	720	900	1080	1260	1440	1620	1800
N	2	2	2	2	2	2	2	2	2	2	2	2	2	2	2
Total Hirschsprung's Disease	1.15 (0.9, 1.4)	1.1 (0.87, 1.33)	1.04 (0.83, 1.25)	0.99 (0.8, 1.18)	0.93 (0.76, 1.11)	0.83 (0.69, 0.96)	0.78 (-0.63, 0.94)	0.45 (0.42, 0.48)	0.47 (0.42, 0.53)	0.18 (0.12, 0.24)	-0.11 (-0.3, 0.07)	-0.24 (-0.46, -0.01)	-0.34 (-0.59, -0.09)	-0.31 (-0.51, -0.1)	-0.18 (-0.3, -0.06)
n	1	1	1	1	1	1	1	1	1	1	1	1	1	1	1
No Intestinal Transplant*	1.65	1.56	1.46	1.37	1.28	1.09	1.09	0.51	0.59	0.05	-0.48	-0.68	-0.84	-0.72	-0.42
n	1	1	1	1	1	1	1	1	1	1	1	1	1	1	1
Intestinal Transplant*	0.65	0.64	0.62	0.61	0.59	0.56	0.47	0.38	0.36	0.31	0.26	0.21	0.16	0.11	0.06

Z-scores expressed as median (Interquartile Range); Shading: No shading – stable growth (+0.5 z-score from baseline), red – deceleration (>-0.5 from baseline); *Median only

Table S7. Weight, Length/Height and Body Mass Index Z-Scores from Parenteral Nutrition Wean (Baseline) up to Five Years Post-Wean in Children with Intestinal Failure (Microvillus Atrophy) who Received an Intestinal Transplant

Days	Baseline Weight	30	60	90	120	180	360	540	720	900	1080	1260	1440	1620	1800
N	4	4	4	4	4	4	3	3	3	3	3	3	2	2	2
Total Microvillus Atrophy	-0.59 (-1.23, 0.3)	-0.47 (-1.3, 0.43)	-0.37 (-1.36, 0.51)	-0.53 (-1.4, 0.28)	-0.75 (-1.45, -0.05)	-1.2 (-1.61, -0.69)	-0.85 (-1.12, -0.11)	-0.41 (-0.92, 0.15)	-0.19 (-0.79, 0.29)	-0.22 (-0.78, 0.25)	-0.29 (-0.79, -0.04)	-0.36 (-0.8, -0.17)	-0.13 (-0.28, 0.01)	-0.09 (-0.29, 0.1)	-0.07 (-0.32, 0.17)

Z-scores expressed as median (Interquartile Range); Shading: No shading – stable growth (+0.5 z-score from baseline), green – acceleration (>+0.5 from baseline)

Days	Baseline Lenght/Height	30	60	90	120	180	360	540	720	900	1080	1260	1440	1620	1800
N	4	4	4	4	4	4	3	3	3	3	3	3	2	2	2
Total Microvillus Atrophy	-2.48 (-3.32, -2.07)	-2.48 (-3.12, -2.08)	-2.47 (-2.99, -2.07)	-2.45 (-2.85, -2.04)	-2.33 (-2.63, -2.05)	-2.4 (-2.65, -2.06)	-1.98 (-2.47, -1.35)	-1.69 (-2.26, -0.92)	-1.3 (-1.88, -0.72)	-1.19 (-1.63, -0.67)	-1.11 (-1.41, -0.68)	-1.03 (-1.19, -0.67)	-0.63 (-0.8, -0.47)	-0.59 (-0.74, -0.44)	-0.51 (-0.66, -0.36)

Z-scores expressed as median (Interquartile Range); Shading: No shading – stable growth (+0.5 z-score from baseline), green – acceleration (>+0.5 from baseline)

Days	Baseline Body Mass Index	30	60	90	120	180	360	540	720	900	1080	1260	1440	1620	1800
N	1	1	1	1	1	1	1	1	1	1	1	1	0	0	0
Total Microvillus Atrophy*	1.23	1.19	1.14	1.1	1.11	1.14	1.21	1.08	0.7	0.32	-0.06	-0.44			

Z-scores expressed as median (Interquartile Range); Shading: No shading – stable growth (+0.5 z-score from baseline), red – deceleration (>-0.5 from baseline); *Median only

Table S8. Weight, Length/Height and Body Mass Index Z-Scores from Parenteral Nutrition Wean (Baseline) up to Five Years Post-Wean in Children with Intestinal Failure (Microvillus Inclusion Disease) who Received an Intestinal Transplant

Days	Baseline Weight	30	60	90	120	180	360	540	720	900	1080	1260	1440	1620	1800
N	2	2	2	2	2	2	2	2	2	1	1	1	1	0	0
Total Microvillus Inclusion Disease*	-1.23 (-1.48, -0.99)	-1.28 (-1.55, -1.02)	-1.34 (-1.61, -1.06)	-1.38 (-1.68, -1.09)	-1.43 (-1.74, -1.11)	-1.52 (-1.87, -1.16)	-1.9 (-2.42, -1.37)	-1.72 (-2.13, -1.3)	-1.37 (-1.59, -1.14)	-0.95	-0.99	-1.02	-1.06		

Z-scores expressed as median (Interquartile range); Shading: No shading – stable growth (+0.5 z-score from baseline), red – deceleration (>-0.5 from baseline); *Median only when n=1

Days	Baseline Length/Height	30	60	90	120	180	360	540	720	900	1080	1260	1440	1620	1800
N	2	2	2	2	2	2	2	2	2	1	1	1	1	0	0
Total Microvillus Inclusion Disease*	-1.67 (-2.02, -1.32)	-1.42 (-1.61, -1.22)	-1.17 (-1.21, -1.14)	-1.13 (-1.15, -1.1)	-1.17 (-1.21, -1.13)	-1.25 (-1.33, -1.17)	-1.66 (-1.93, -1.4)	-1.61 (-1.83, -1.39)	-1.4 (-1.49, -1.31)	-1.26	-1.3	-1.34	-1.38		

Z-scores expressed as median (Interquartile range); No shading – stable growth (+0.5 z-score from baseline), green – acceleration (>+0.5 from baseline); *Median only when n=1

Days	Baseline Body Mass Index	30	60	90	120	180	360	540	720	900	1080	1260	1440	1620	1800
N	2	2	2	2	2	2	2	2	2	1	1	1	1	0	0
Total Microvillus Inclusion Disease*	-0.13 (-0.84, 0.59)	-0.48 (-1.05, 0.09)	-0.82 (-1.25, -0.4)	-0.94 (-1.34, -0.55)	-0.97 (-1.38, -0.57)	-1.03 (-1.46, -0.6)	-1.2 (-1.71, -0.69)	-1.03 (-1.46, -0.61)	-0.77 (-1.06, -0.48)	-0.19	-0.2	-0.21	-0.22		

Z-scores expressed as median (Interquartile range); No shading – stable growth (+0.5 z-score from baseline), red – deceleration (>-0.5 from baseline); *Median only when n=1

Table S9. Weight, Length/Height and Body Mass Index Z-Scores from Parenteral Nutrition Wean (Baseline) up to Five Years Post-Wean in Children with Intestinal Failure (Tufting Enteropathy) who Received an Intestinal Transplant

Days	Baseline Weight	30	60	90	120	180	360	540	720	900	1080	1260	1440	1620	1800
N	1	1	1	1	1	1	1	1	1	1	1	1	1	1	1
Total Pseudo-obstruction	-1	-1	-1	-1	-0.99	-0.99	-0.98	-1.29	-1.6	-1.57	-1.54	-1.5	-1.22	-0.87	-0.69

Z-scores expressed as median; No shading – stable growth (+0.5 z-score from baseline), red – deceleration (>-0.5 from baseline)

Days	Baseline Length/Height	30	60	90	120	180	360	540	720	900	1080	1260	1440	1620	1800
N	1	1	1	1	1	1	1	1	1	1	1	1	1	1	1
Total Pseudo-obstruction	-1.25	-1.25	-1.25	-1.26	-1.26	-1.26	-1.28	-1.28	-1.28	-1.26	-1.23	-1.21	-0.5	0.43	0.43

Z-scores expressed as median; No shading – stable growth (+0.5 z-score from baseline), green – acceleration (>+0.5 from baseline)

Days	Baseline Body Mass Index	30	60	90	120	180	360	540	720	900	1080	1260	1440	1620	1800
N	1	1	1	1	1	1	1	1	1	1	1	1	1	1	1
Total Pseudo-obstruction	-0.24	-0.23	-0.23	-0.22	-0.22	-0.21	-0.17	-0.67	-1.19	-1.21	-1.22	-1.22	-1.33	-1.47	-1.19

Z-scores expressed as median; No shading – stable growth (+0.5 z-score from baseline), red – deceleration (>-0.5 from baseline)

Table S10. Weight and Length/Height Z-Scores from Parenteral Nutrition Wean (Baseline) up to Five Years Post-Wean in Children with Intestinal Failure (Tufting Enteropathy) who Received an Intestinal Transplant

Days	Baseline Weight	30	60	90	120	180	360	540	720	900	1080	1260	1440	1620	1800
N	1	1	1	1	1	1	0	0	0	0	0	0	0	0	0
Total Tufting Enteropathy	-0.37	-1.19	-1.57	-1.77	-1.97	-2.37									

Z-scores expressed as median; No shading – stable growth (+0.5 z-score from baseline), red – deceleration (>-0.5 from baseline)

Days	Baseline Length/Height	30	60	90	120	180	360	540	720	900	1080	1260	1440	1620	1800
N	1	1	1	1	1	1	0	0	0	0	0	0	0	0	0
Total Tufting Enteropathy	-2.18	-1.97	-1.96	-2.03	-2.1	-2.23									

Z-scores expressed as median; No shading – stable growth (+0.5 z-score from baseline)
